# A new species of Crocodile Newt,genus *Tylototriton* (Amphibia,Caudata,Salamandridae) from the mountains of Kachin State,northern Myanmar

**DOI:** 10.24272/j.issn.2095-8137.2019.043

**Published:** 2019-05-18

**Authors:** Than Zaw, Paw Lay, Parinya Pawangkhanant, Vladislav A. Gorin, Nikolay A. Poyarkov

**Affiliations:** 1 1Zoology Department, Mohnyin Degree College, Mohnyin, Kachin State, Myanmar; 2 Flora and Fauna International, Lon Ton Village, Indawgyi, Kachin State, Myanmar; 3 Bansomdejchaopraya Rajabhat University, Hiran Ruchi, Thon Buri, Bangkok 10600, Thailand; 4 Department of Vertebrate Zoology, Biological Faculty, Lomonosov Moscow State University, Moscow 119234, Russia; 5 Joint Russian-Vietnamese Tropical Research and Technological Center, Nghia Do, Cau Giay, Hanoi, Vietnam

**Keywords:** *Tylototriton kachinorum ***sp. nov.**, mtDNA genealogy, ND2, 16S rRNA, Shan, Biogeography, Endemism, Taxonomy

## Abstract

We describe a new species of the genus *Tylototriton* from Ingyin Taung Mt., Mohnyin Township, Kachin State, Myanmar, based on morphological and molecular evidence. The new species is assigned to the subgenus *Tylototriton* s. str. and is clearly distinct from all known congeners by the following characters: medium body size; thin, long tail, lacking lateral grooves; rough skin; truncate snout; wide, protruding supratemporal bony ridges on head, beginning at anterior corner of orbit; weak, almost indistinct sagittal ridge; long, thin limbs, broadly overlapping when adpressed along body; distinct, wide, non-segmented vertebral ridge; 13 or 14 rib nodules; brown to dark-brown background coloration with dull orange-brown to yellowish-brown markings on labial regions, parotoids, rib nodules, whole limbs, vent, and ventral tail ridge. We also briefly discuss biogeography and species diversity of the genus *Tylototriton* in Myanmar.

A new species of Crocodile Newt, genus *Tylototriton* (Amphibia, Caudata, Salamandridae) from the mountains of Kachin State, northern Myanmar

## INTRODUCTION

The salamandrid genus *Tylototriton* Anderson, 1871, or Crocodile Newts, currently includes 24 recognized species, inhabiting montane forest areas throughout the Asian monsoon climate zone from eastern Himalaya, southern and central China including Hainan Island, to northern Indochina including Vietnam, Laos, Thailand, and Myanmar ( Hernandez, 2016; Wang et al., 2018). Recent progress in phylogenetic studies of the genus *Tylototriton* has indicated that the genus is monophyletic ( Nishikawa et al., 2013a, 2013b; Phimmachak et al., 2015; Wang et al., 2018) and includes two major groups, corresponding to the subgenera *Tylototriton* s. str. and *Yaotriton* (Wang et al., 2018). Molecular taxonomy methods have proven to be useful for deciphering taxonomic diversity of the genus *Tylototriton*, with 13 species (over 50%) described in the past five years (Grismer et al., 2018a; Hou et al., 2012; Khatiwada et al., 2015; Nishikawa et al., 2013a, 2013b, 2014; Phimmachak et al., 2015; Shen et al., 2012; Yang et al., 2014; Zhao et al., 2012). However, recent molecular surveys indicate that our knowledge on taxonomic diversity of the genus *Tylototriton* is still far from complete, revealing several cryptic lineages likely corresponding to as yet undescribed species (Grismer et al., 2018a;Wang et al., 2018).

Myanmar, previously known as Burma, is the largest country of mainland Southeast Asia. Despite this, its herpetofauna remains one of the least explored in the region (Grismer et al., 2018a). Members of the genus *Tylototriton* have long been recorded from northern and eastern parts of Myanmar and have been traditionally classified as *T. verrucosus* Anderson, 1871 (Gyi, 1969). Nishikawa et al. (2014), based on the examination of specimens assigned to *T. verrucosus* collected from the Shan Plateau in eastern Myanmar and pet-trade animals assumed to originate from Myanmar, recently described a new species, *T. shanorum* Nishikawa, Matsui & Rao, 2014. Soon after, Phimmachak et al.(2015） published sequence data for specimens collected from the Sagaing Region and Kachin State in northern Myanmar, which were reported as *T. verrucosus*. More recently, Grismer et al. (2018a) demonstrated the presence of two morphologically and genetically distinct lineages of *Tylototriton* in the Shan Plateau and described a new species from its north-western edge, *T. ngarsuensis* Grismer, Wood, Quah, Thura, Espinoza, Grismer, Murdoch & Lin, 2018. The same work of Grismer et al. (2018a) reanalyzed sequences of specimens from the Sagaing and Kachin regions of Myanmar and demonstrated that they belong to a distinct lineage – *Tylototriton* sp. 1, distinct from *T. verrucosus* s. str. Recent work also indicated the presence of *T. himalayanus* Khatiwada, Wang, Ghimire, Vasudevan, Paudel & Jiang, 2015 (a species described from Nepalese Himalaya) in northern Myanmar, but without providing voucher specimen information or any other justification for this identification (Hernandez, 2016; Hernandez, 2018). The recent monographic review of the genus *Tylototriton* by Hernandez (2016) also indicated the possibility of the occurrence of *T. uyenoi* Nishikawa, Khonsue, Pomchote & Matsui, 2013 and *T. shanjing* Nussbaum, Brodie & Yang, 1995 in parts of Myanmar adjacent to northern Thailand and the southwestern Yunnan Province of China; however, these records are not supported by voucher specimens. Thus, our knowledge on the taxonomic composition and diversity of the genus *Tylototriton* in Myanmar is still far from complete.

In the present paper, we report on a new population of the genus *Tylototriton* from the Kachin Hills in the southern part of Kachin State, northern Myanmar. We applied morphological and molecular methods to evaluate its taxonomic status and describe it as a new species. We also discuss biogeography and taxonomy of the genus *Tylototriton* in Myanmar.

## MATERIALS AND METHODS

### Sample collection

**Figure 1:**
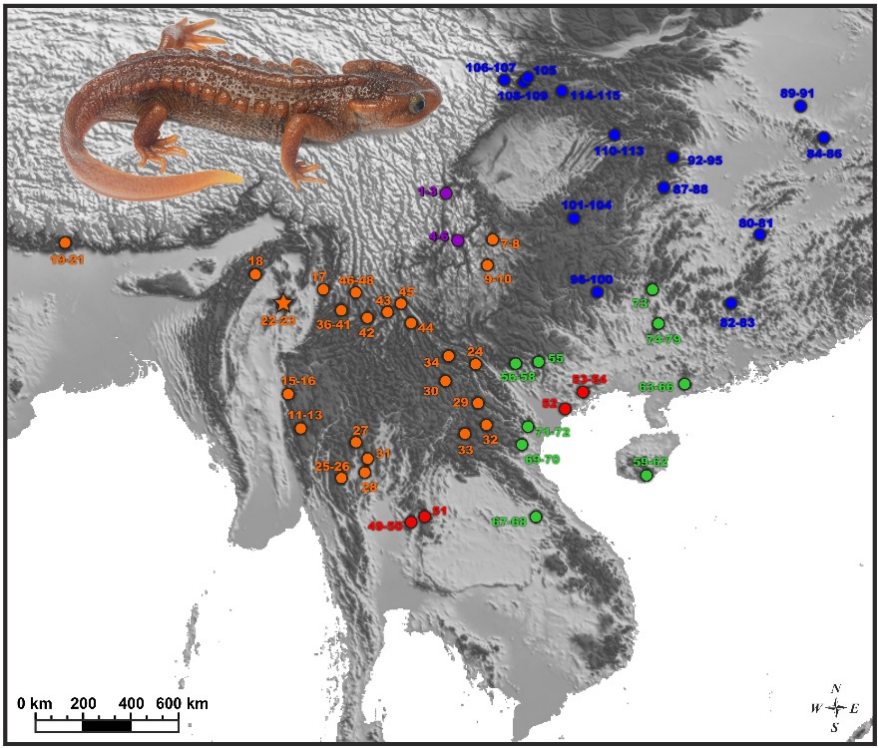
Distribution of *Tylototriton* and sampling localities examined in this study

Fieldwork was carried out in northern Myanmar, Kachin State, from 14 to 21 July 2018. Specimens of *Tylototriton* sp. were collected by hand in swamps in forest clearings surrounded by montane evergreen tropical forests of Ingyin Taung Mountain, Indawgyi Lake area, Kachin State (Figure 1; samples 22–23). Geographic coordinates and altitude were obtained using a Garmin GPSMAP 60CSx GPS receiver (Garmin Ltd., USA) and recorded in datum WGS 84. Specimens were euthanized by 20% benzocaine and tissue samples for genetic analysis were taken and stored in 96% ethanol (femoral muscles) prior to preservation. Specimens were subsequently preserved in 70% ethanol and deposited in the herpetological collection of the Zoological Museum of Moscow State University (ZMMU) in Moscow, Russia; Zoological Institute of Russian Academy of Sciences (ZISP) in St. Petersburg, Russia; and Zoology Department of University of Mandalay (ZDUM), Mandalay, Myanmar.

**Figure 2:**
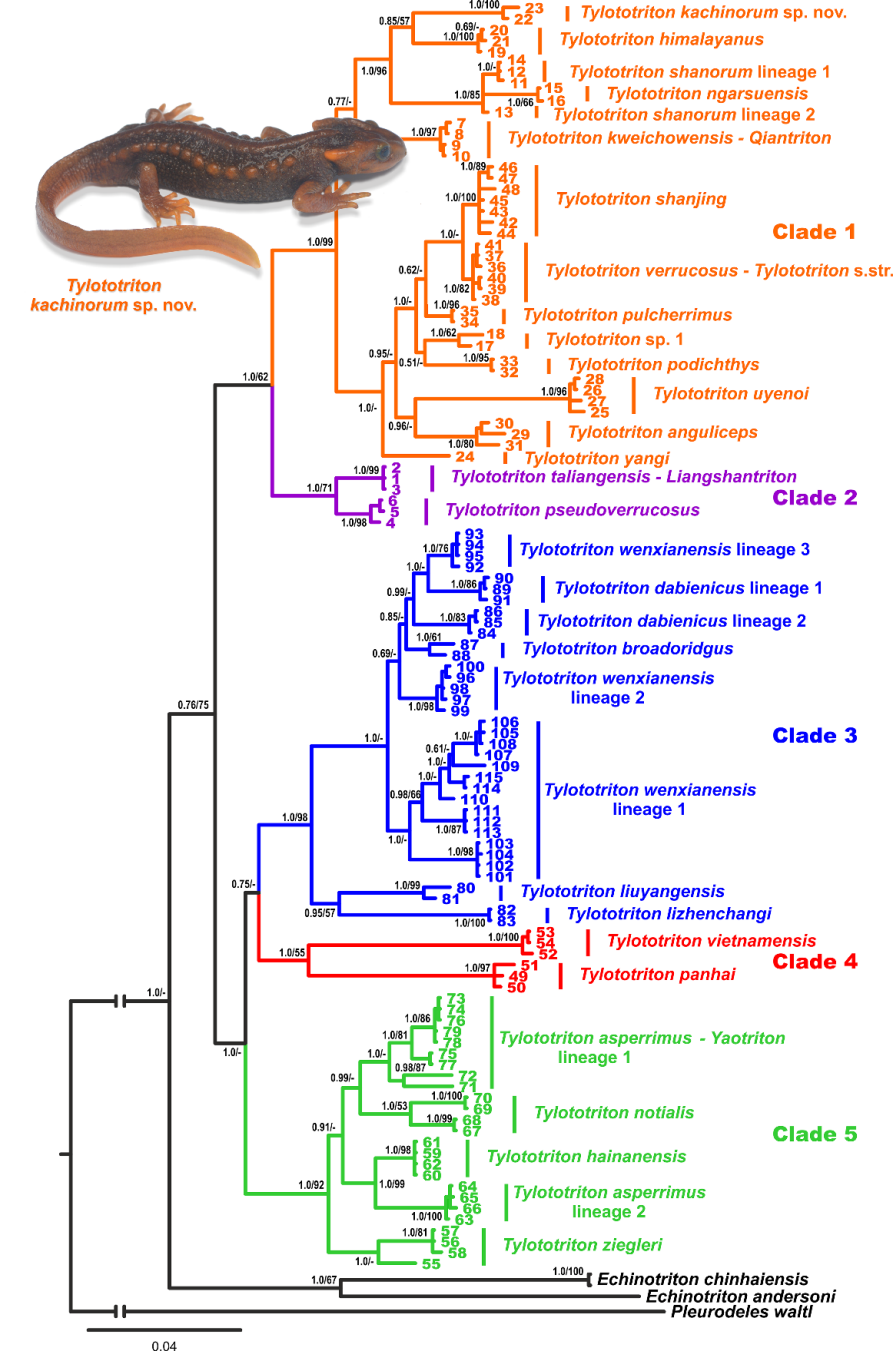
Bayesian inference consensus tree of genus *Tylototriton* derived from analysis of 1 157 bp *ND2* and 508 bp 16S rRNA gene fragments

Colors of dots correspond to clades 1–5 denoted in Figure 2. Sample numbers 1–115 refer to Table 1. Type locality of new species *Tylototriton kachinorum* **sp. nov.** is marked with a star. Photo by Nikolay A. Poyarkov.

### Morphological description

Specimens of *Tylototriton* sp. were photographed in life and after preservation. The sex and maturity of the specimens and number of eggs were checked and counted by minor dissections. Measurements were taken using a digital caliper to the nearest 0.01 mm, subsequently rounded to 0.1 mm. We used a stereoscopic light binocular microscope when necessary. Statistical analyses were performed with Statistica 8.0 (Version 8.0; StatSoft, Tulsa, OK, USA).

### Adult morphology

Morphometrics followed Nishikawa et al. (2014), Khatiwada et al. (2015), and Okamiya et al. (2018) and included the following 24 measurements: (1) SVL (snout-vent length) from tip of snout to anterior tip of vent; (2) HL (head length); (3) HW (head width); (4) MXHW (maximum head width); (5) IND (internarial distance); (6) AGD (axilla-groin distance); (7) TRL (trunk length); (8) TAL (tail length) from anterior tip of vent to tail tip; (9) VL (vent length); (10) FLL (forelimb length); (11) HLL (hindlimb length); (12) VTW (vomerine tooth series width): greatest width of vomerine tooth series; (13) VTL (vomerine tooth series length): greatest length of vomerine tooth series; (14) LJL (lower jaw length from tip of lower jaw to articulation of upper and lower jaws); (15) SL (snout length from tip of snout to anterior tip of upper eyelid); (16) IOD (minimum interorbital distance); (17) UEW (maximum upper eyelid width); (18) UEL (upper eyelid length, distance between anterior and posterior angles); (19) OL (orbit length); (20) BTAW (basal tail width at level of anterior tip of cloaca); (21) MTAW (tail width at mid-level of tail); (22) MXTAH (maximum tail height); (23) MTAH (tail height at mid-level of tail); (24) ON (orbitonarial distance). For holotype description, we examined the following 12 morphometric and three meristic characters following Poyarkov et al. (2012) and Okamiya et al. (2018): additional morphometric characters: (25) ICD (intercanthal distance); (26) CW (chest width); (27) NSD (nostril-snout distance); (28) 1FL (first finger length from base to tip); (29) 2FL (second finger length from base to tip); (30) 3FL (third finger length from base to tip); (31) 4FL (fourth finger length from base to tip); (32) 1TL (first toe length from base to tip); (33) 2TL (second toe length from base to tip); (34) 3TL (third toe length from base to tip); (35) 4TL (fourth toe length from base to tip); (36) 5TL (fifth toe length from base to tip); meristic characters: (37) UJTN (number of teeth on upper jaw); (38) LJTN (number of teeth on lower jaw); (39) VTN, number of teeth on vomer. We also recorded the following characters as per Nishikawa et al. (2014) and Grismer et al. (2018a): shape of vomerine teeth and their positional relationship relative to choanae; skin texture; number and shape of rib nodules counted from posterior margin of vent to axilla; width and prominence of vertebral ridge and head ridges; and coloration of dorsum, venter, head, labial region, parotoid glands, rib nodules, limbs, soles, palms, tail surfaces, and vent region.

The characters of adult morphology chosen for comparison and data on other *Tylototriton* species were taken from the following sources: Anderson (1871); Böhme et al. (2005); Chen et al. (2010); Fang & Chang (1932); Fei et al. (1984); Grismer et al. (2018a); Hernandez (2016); Hou et al. (2012); Khatiwada et al. (2015); Le et al. (2015); Liu (1950); Nishikawa et al. (2013a; 2013b; 2014); Nussbaum et al. (1995); Phimmachak et al. (2015); Qian et al. (2017); Shen et al. (2012); Stuart et al. (2010); Unterstein (1930); Yang et al. (2014).

### Larval morphology

Description of larval morphology followed Okamiya et al. (2018). For larval specimens, we recorded nine morphometric characters including (1) SVL, (2) HL, (3) HW, (4) OL, (5) AGD, (6) TAL, (7) FLL, (8) HLL, and (9) MXTAH (definition same as for adult morphology). Developmental stages were determined following Grosse (2013).

### DNA isolation, PCR, and sequencing

Total genomic DNA was extracted from 95% ethanol-preserved muscle tissues using standard phenol-chloroform extraction protocols (Hillis et al., 1996). Total DNA concentration was estimated in 1 μL using a NanoDrop 2000 spectrophotometer (Thermo Scientific, USA), and consequently adjusted to 100 ng DNA/μL.

We amplified two mtDNA fragments consisting of partial sequences of the *ND2* and 16S rRNA mtDNA genes. These markers were chosen as they are useful in studies of *Tylototriton* phylogeny and taxonomy (Nishikawa et al., 2013a, 2013b, 2014;Wang et al., 2018; Zhao et al., 2012 and references therein). We used the 16L-1 (forward) (5'-CTGACCGTGCAAA GGTAGCGTAATCACT-3') and 16H-1 (reverse) (5'-CTCCGG TCTGAACTCAGATCACGTAGG-3') primers to amplify the 16S rRNA fragments (Hedges, 1994). For amplification and sequencing of the *ND2* gene, we used the SL-1 (forward) (5'-ATAGAGGTTCAAACCCTCTC-3') and SL-2 (reverse) (5'-TTAAAGTGTCTGGGTTGCATTCAG-3') primers of Wang et al. (2018). Polymerase chain reaction (PCR) was performed in 20 μL reactions using 50 ng genomic DNA, 10 nmol of each primer, 15 nmol of each dNTP, 50 nmol additional MgCl_2_, Taq PCR buffer (10 mmol/L Tris-HCl, pH 8.3, 50 mmol/L KCl, 1.1 mmol/L MgCl_2_, and 0.01% gelatin), and 1 U of Taq DNA polymerase. PCR cycles included an initial denaturation step of 4 min at 94 °C and 35 cycles of denaturation for 30 s at 94 °C, primer annealing for 30 s at 48–58 °C, and extension for 1 min 30 s at 72 °C. PCR products were visualized by agarose gel electrophoresis in the presence of ethidium bromide and consequently purified using 2 μL from a 1:4 dilution of ExoSapIt (Amersham, UK) per 5 μL of PCR product prior to cycle sequencing. Sequencing was performed in both directions using the same primers as used in PCR on an ABI3730xl automated sequencer (Applied Biosystems, USA) at Evrogen Inc., Moscow (Russia). The newly obtained sequences were aligned and deposited in GenBank under the accession numbers MK095616–MK095619 and MK097271–MK097274 (Table 1). Sequences of 25 other *Tylototriton* species used for comparisons were obtained from GenBank (Table 1).

**Table 1:** Sequences and voucher specimens of *Tylototriton* and outgroup taxa used in this study

**Sample No.**	**Species name**	**Voucher number**	**Locality**	**16S rRNA**	***ND2***
	**Ingroup:**				
1	*Tylototriton taliangensis*	CIB GG200110183	Shimian Co., Ya'an City, Sichuan, China	KY800559	KC147819
2	*Tylototriton taliangensis*	CIB GG200110185	Shimian Co., Ya'an City, Sichuan, China	KY800560	KY800829
3	*Tylototriton taliangensis*	CIB GG200110186	Shimian Co. Ya'an City, Sichuan, China	KY800561	KY800830
4	*Tylototriton pseudoverrucosus*	CIB WCG2012003	Ningnan Co., Liangshan Yi Autonomous Prefecture, Sichuan, China	KY800597	KY800861
5	*Tylototriton pseudoverrucosus*	CIB WCG2012007	Ningnan Co., Liangshan Yi Autonomous Prefecture, Sichuan, China	KY800598	KY800862
6	*Tylototriton pseudoverrucosus*	CIB WCG2012012	Ningnan Co., Liangshan Yi Autonomous Prefecture, Sichuan, China	KY800599	KY800860
7	*Tylototriton kweichowensis*	CIB WG20080818014	Bijie City, Guizhou, China	KY800551	KY800823
8	*Tylototriton kweichowensis*	CIB WG20080818018	Bijie City, Guizhou, China	KY800552	KY800824
9	*Tylototriton kweichowensis*	CIB 20050213	Shuicheng Co., Guizhou, China	KY800557	KY800827
10	*Tylototriton kweichowensis*	CIB 20050215	Shuicheng Co., Guizhou, China	KY800558	KY800828
11	*Tylototriton shanorum* lineage 1	CAS 230933	Taunggyi Township, Shan, Myanmar	—	AB922822
12	*Tylototriton shanorum* lineage 1	CAS 230940	Taunggyi Township, Shan, Myanmar	—	AB922823
13	*Tylototriton shanorum* lineage 1	CAS 230899	Taunggyi Township, Shan, Myanmar	—	HM770087
14	*Tylototriton shanorum* lineage 2	KUHE 42348	pet trade, presumably from Myanmar	—	AB769544
15	*Tylototriton ngarsuensis*	LSUHC 13762	Ngar Su, Shan, Myanmar	—	MH836585
16	*Tylototriton ngarsuensis*	LSUHC 13763	Ngar Su, Shan, Myanmar	—	MH836584
17	*Tylototriton* sp. 1	CAS 245418	Chipwi Township, Kachin State, Myanmar	—	KT304279
18	*Tylototriton* sp. 1	CAS 245290	Lahe Township, Sagaing Region, Myanmar	—	KT304278
19	*Tylototriton himalayanus*	CIB 201406246	Mai Pokhari, Illam, Mechi, Nepal	KY800590	KT765173
20	*Tylototriton himalayanus*	CIB 201406284	Mai Pokhari, Illam, Mechi, Nepal	KY800591	KT765207
21	*Tylototriton himalayanus*	CIB 201406285	Mai Pokhari, Illam, Mechi, Nepal	KY800592	KT765208
22	*Tylototriton kachinorum * **sp. nov.**	ZMMU A5953	Ingyin Taung Mt., Indawgyi, Kachin, Myanmar	MK095618	MK097273
23	*Tylototriton kachinorum * **sp. nov.**	ZDUM-0103	Ingyin Taung Mt., Indawgyi, Kachin, Myanmar	MK095619	MK097274
24	*Tylototriton yangi*	KUHE 42282	Pingbian Co., Yunnan, China	KY800624	KY800887
25	*Tylototriton uyenoi*	ZMMU NAP-08220	Doi Inthanon, Chiang Mai, Thailand	MK095617	MK097272
26	*Tylototriton uyenoi*	KUHE 19037	Doi Inthanon, Chiang Mai, Thailand	—	AB830730
27	*Tylototriton uyenoi*	KUHE 19147	Doi Ang Khang, Chiang Mai, Thailand	—	AB830729
28	*Tylototriton uyenoi*	ITNT1	Doi Suthep, Chiang Mai, Thailand	—	AB830733
29	*Tylototriton anguliceps*	TBU PAE671	Thuan Chau, Son La, Vietnam	—	LC017833
30	*Tylototriton anguliceps*	VNMN A20143	Muong Nhe, Dien Bien, Vietnam	—	LC017832
31	*Tylototriton anguliceps*	LK003	Doi Lahnga, Chiang Mai, Thailand	—	AB830728
32	*Tylototriton podichthys*	KUHE 34399	Phu Pan, Xam Neua, Laos	—	AB830727
33	*Tylototriton podichthys*	IEBR A2014-1	Xam Neua, Huaphanh, Laos	—	LC017835
34	*Tylototriton pulcherrimus*	CIB TY 040	Lüchun Co., Yunnan, China	KY800626	KY800890
35	*Tylototriton pulcherrimus*	KUHE 46406	Pet Trade	KY800620	KY800880
36	*Tylototriton verrucosus*	CIB TSHS1	Longchuan Co., Dehong, Yunnan, China	KY800581	KY800847
37	*Tylototriton verrucosus*	CIB-TSHS2	Longchuan Co., Dehong, Yunnan, China	KY800582	KY800848
38	*Tylototriton verrucosus*	CIB TSHS3	Longchuan Co., Dehong, Yunnan, China	KY800583	KY800849
39	*Tylototriton verrucosus*	CIB TSHS4	Longchuan Co., Dehong, Yunnan, China	KY800584	KY800850
40	*Tylototriton verrucosus*	CIB TSHS5	Longchuan Co., Dehong, Yunnan, China	KY800585	KY800851
41	*Tylototriton verrucosus*	CIB TSHS6	Longchuan Co., Dehong, Yunnan, China	KY800586	KY800852
42	*Tylototriton shanjing*	KIZ 201306081	Yongde Co., Yunnan, China	KY800593	KY800856
43	*Tylototriton shanjing*	KIZ 201306098	Yun Co., Yunnan, China	KY800594	KY800857
44	*Tylototriton shanjing*	KIZ 201306102	Jingdong Co., Yunnan, China	KY800595	KY800858
45	*Tylototriton shanjing*	KIZ 201306108	Nanjian Co., Yunnan, China	KY800596	KY800859
46	*Tylototriton shanjing*	CIB 980004	Baoshan City, Yunnan, China	KY800562	KY800831
47	*Tylototriton shanjing*	CIB 980005	Baoshan City, Yunnan, China	KY800563	KY800832
48	*Tylototriton shanjing*	CIB 980006	Baoshan City, Yunnan, China	KY800564	KY800833
49	*Tylototriton panhai*	ZMMU NAP-08217	Phu Hin Rong Kla NP, Phitsanulok, Thailand	MK095616	MK097271
50	*Tylototriton panhai*	PH019	Phu Hin Rong Kla NP, Phitsanulok, Thailand	—	AB830735
51	*Tylototriton panhai*	PL009	Phu Luang WS, Loei, Thailand	—	AB830736
52	*Tylototriton vietnamensis*	IEBR A.3674	Yen Tu, Bac Giang, Vietnam	KY800614	KY800874
53	*Tylototriton vietnamensis*	IEBR A.0702	Mauson, Lang Son, Vietnam	KY800612	KY800872
54	*Tylototriton vietnamensis*	IEBR A.0701	Mauson, Lang Son, Vietnam	KY800613	KY800873
55	*Tylototriton ziegleri*	VNMN 3389	Bao Lac, Cao Bang, Vietnam	—	KY800888
56	*Tylototriton ziegleri*	VNMN 3390	Quan Ba, Ha Giang, Vietnam	KY800625	KY800889
57	*Tylototriton ziegleri*	VNUH HG.082	Quan Ba, Ha Giang, Vietnam	KY800610	KY800870
58	*Tylototriton ziegleri*	VNUH HG.081	Quan Ba, Ha Giang, Vietnam	KY800611	KY800871
59	*Tylototriton hainanensis*	CIB 20081048	Mt. Diaoluo, Hainan, China	KY800553	KC147817
60	*Tylototriton hainanensis*	CIB 20081049	Mt. Diaoluo, Hainan, China	KY800554	KC147818
61	*Tylototriton hainanensis*	CIB 20081051	Mt. Diaoluo, Hainan, China	KY800555	KY800825
62	*Tylototriton hainanensis*	CIB 20081052	Mt. Diaoluo, Hainan, China	KY800556	KY800826
63	*Tylototriton asperrimus* lineage 2	CIB XZ20091201	Xinyi City, Guangdong, China	KY800616	KY800876
64	*Tylototriton asperrimus* lineage 2	CIB XZ20091204	Xinyi City, Guangdong, China	KY800619	KY800879
65	*Tylototriton asperrimus* lineage 2	CIB XZ20091	Xinyi City, Guangdong, China	KY800618	KY800878
66	*Tylototriton asperrimus* lineage 2	CIB XZ20092	Xinyi City, Guangdong, China	KY800617	KY800877
67	*Tylototriton notialis*	FMNH HERP271120	Boualapha Dist., Khammouan, Laos	—	HM462061
68	*Tylototriton notialis*	FMNH HERP271121	Boualapha Dist., Khammouan, Laos	—	HM462062
69	*Tylototriton notialis*	VNMN TAO1229	Pu Hoat, Nghe An, Vietnam	—	KY800883
70	*Tylototriton notialis*	VNMN TAO1235	Pu Hoat, Nghe An, Vietnam	—	KY800884
71	*Tylototriton asperrimus* lineage 1	VNMN TAO1213	Thuong Tien, Hoa Binh, Vietnam	KY800623	KY800885
72	*Tylototriton asperrimus* lineage 1	VNMN TAO1214	Thuong Tien, Hoa Binh, Vietnam	—	KY800886
73	*Tylototriton asperrimus* lineage 1	CIB 70063	Longsheng Co., Guangxi, China	KY800549	KC147816
74	*Tylototriton asperrimus* lineage 1	CIB GX20080714	Jinxiu Co., Guangxi, China	KY800546	KY800819
75	*Tylototriton asperrimus* lineage 1	CIB GX200807010	Jinxiu Co., Guangxi, China	KY800547	KY800820
76	*Tylototriton asperrimus* lineage 1	CIB GX200807012	Jinxiu Co., Guangxi, China	KY800548	KY800821
77	*Tylototriton asperrimus* lineage 1	CIB GX200807016	Jinxiu Co., Guangxi, China	KY800550	KY800822
78	*Tylototriton asperrimus* lineage 1	CIB 20070715	Jinxiu Co., Guangxi, China	KY800565	KY800834
79	*Tylototriton asperrimus* lineage 1	CIB 200807055	Jinxiu Co., Guangxi, China	KY800566	KC147815
80	*Tylototriton liuyangensis*	CSUFT 20100108	Liuyang City, Hunan, China	KY800606	KJ205598
81	*Tylototriton liuyangensis*	CIB 110601F06	Liuyang City, Hunan, China	KY800615	KY800875
82	*Tylototriton lizhenchangi*	KUHE 42316	Yizhang Co., Hunan, China	KY800621	KY800881
83	*Tylototriton lizhenchangi*	KUHE 42317	Yizhang Co., Hunan, China	KY800622	KY800882
84	*Tylototriton dabienicus* lineage 2	CIB 08042905-2	Yuexi Co. Anhui, China	KY800587	KY800853
85	*Tylototriton dabienicus* lineage 2	CIB 08042905-3	Yuexi Co. Anhui, China	KY800588	KY800854
86	*Tylototriton dabienicus* lineage 2	CIB 08042905-4	Yuexi Co. Anhui, China	KY800589	KY800855
87	*Tylototriton broadoridgus*	CIB 200085	Sangzhi Co., Hunan, China	KY800569	KC147814
88	*Tylototriton broadoridgus*	CIB 200084	Sangzhi Co., Hunan, China	KY800570	KY800837
89	*Tylototriton dabienicus* lineage 1	HNNU 1004-015	Shangcheng Co., Anhui, China	KY800607	KC147811
90	*Tylototriton dabienicus* lineage 1	HNNU 1004-024	Shangcheng Co., Anhui, China	KY800608	KC147812
91	*Tylototriton dabienicus* lineage 1	HNNU 1004-026	Shangcheng Co., Anhui, China	KY800609	KY800869
92	*Tylototriton wenxianensis* lineage 3	CIB WH10001	Wufeng Co., Hubei, China	KY800600	KY800863
93	*Tylototriton wenxianensis* lineage 3	CIB WH10002	Wufeng Co., Hubei, China	KY800601	KY800864
94	*Tylototriton wenxianensis* lineage 3	CIB WH10003	Wufeng Co., Hubei, China	KY800602	KY800865
95	*Tylototriton wenxianensis* lineage 3	CIB WH10007	Wufeng Co., Hubei, China	KY800603	KY800866
96	*Tylototriton wenxianensis* lineage 2	CIB Wg20090730001	Libo Co., Guizhou, China	KY800575	KY800842
97	*Tylototriton wenxianensis* lineage 2	CIB Wg20090730002	Libo Co., Guizhou, China	KY800576	KY800843
98	*Tylototriton wenxianensis* lineage 2	CIB Wg20090730003	Libo Co., Guizhou, China	KY800577	KY800844
99	*Tylototriton wenxianensis* lineage 2	CIB Wg20090730005	Libo Co., Guizhou, China	KY800578	KY800845
100	*Tylototriton wenxianensis* lineage 2	CIB Wg20090730004	Libo Co., Guizhou, China	KY800580	KY800846
101	*Tylototriton wenxianensis* lineage 1	CIB WG200600019	Suiyang Co., Zunyi, Guizhou, China	KY800544	KY800817
102	*Tylototriton wenxianensis* lineage 1	CIB WG20060007	Suiyang Co., Zunyi, Guizhou, China	KY800545	KY800818
103	*Tylototriton wenxianensis* lineage 1	CIB WG20090601002	Suiyang Co., Zunyi, Guizhou, China	KY800573	KY800840
104	*Tylototriton wenxianensis* lineage 1	CIB WG20090601001	Suiyang Co., Zunyi, Guizhou, China	KY800574	KY800841
105	*Tylototriton wenxianensis* lineage 1	CIB 20090527	Wenxian Co., Gansu, China	KY800579	KC147813
106	*Tylototriton wenxianensis* lineage 1	CIB 2010123101	Pingwu Co., Gansu, China	KY800604	KY800867
107	*Tylototriton wenxianensis* lineage 1	CIB 2010123102	Pingwu Co., Gansu, China	KY800605	KY800868
108	*Tylototriton wenxianensis* lineage 1	CIB 20070639	Qingchuan Co., Sichuan, China	KY800542	KY800815
109	*Tylototriton wenxianensis* lineage 1	CIB 20070638	Qingchuan Co., Sichuan, China	KY800543	KY800816
110	*Tylototriton wenxianensis* lineage 1	CIB 20080002	Yunyang Co., Chongqing, China	KY800540	KY800813
111	*Tylototriton wenxianensis* lineage 1	CIB 20080003	Yunyang Co., Chongqing, China	KY800541	KY800814
112	*Tylototriton wenxianensis* lineage 1	CIB 20081201	Yunyang Co., Chongqing, China	KY800567	KY800835
113	*Tylototriton wenxianensis* lineage 1	CIB 20081202	Yunyang Co., Chongqing, China	KY800568	KY800836
114	*Tylototriton wenxianensis* lineage 1	CIB WA20090601	Wangcang Co., Sichuan, China	KY800571	KY800838
115	*Tylototriton wenxianensis* lineage 1	CIB WA20090602	Wangcang Co., Sichuan, China	KY800572	KY800839
	**Outgroup:**				
116	*Echinotriton chinhaiensis*	CIB ZHJY1	Zhenhai Co., Zhejiang, China	KY800627	KY800891
117	*Echinotriton chinhaiensis*	CIB ZHJY2	Zhenhai Co., Zhejiang, China	KY800628	KY800892
118	*Echinotriton andersoni*	MVZ 232187	Tokunoshima, Kagoshima, Japan	EU880314	EU880314
119	*Pleurodeles waltl*	MVZ 231894	Cadiz, Andalusia, Spain	EU880330	EU880330

For sampling localities see Figure 1. Institutional abbreviations: CAS: California Academy of Sciences, Department of Herpetology, USA; CIB: Chengdu Institute of Biology, Chinese Academy of Sciences, China; CSUFT: Central South University of Forestry and Technology, China; FMNH: Field Museum, USA; HNNU: Henan Normal University, China; IEBR: Institute of Ecology and Biological Resources, Vietnam; KIZ: Kunming Institute of Zoology, Chinese Academy of Sciences, China; KUHE: Graduate School of Human and Environmental Studies of Kyoto University, Japan; LSUHC: La Sierra University, Herpetological Collection, USA; MVZ: Museum of Vertebrate Zoology, University of California, USA; TBU: Thuan Chau University, Vietnam; VNMN: Vietnam National Museum of Nature, Vietnam; VNUH: Vietnam National University Hanoi, Vietnam; ZDUM: Zoological Department, University of Mandalay, Myanmar; ZISP: Zoological Institute, Russian Academy of Sciences, Russia; ZMMU: Zoological Museum of Lomonosov Moscow State University, Russia. Co.: County. —: Not available.

### Phylogenetic analyses

Sequences of partial fragments of *ND2* and 16S rRNA mtDNA for 119 Salamandridae specimens, including 115 representatives of *Tylototriton* (26 species) and four sequences of outgroup members of Salamandridae (*Echinotriton* and *Pleurodeles*) were included in the final alignment with a total length of up to 1 665 bp. Information on voucher specimens and GenBank accession Nos. used in phylogenetic analyses is summarized in Table 1. Nucleotide sequences were initially aligned in MAFFT v.6 (Katoh et al., 2002) with default parameters, and then checked by eye in BioEdit 7.0.5.2 (Hall, 1999) and slightly adjusted.

The dataset was divided into four partitions: three codon-partitions for the *ND2* gene and a single partition for 16S rRNA, with the optimal evolutionary models for each estimated using MODELTEST v.3.06 (Posada & Crandall, 1998). According to the Akaike information criterion (AIC), the TVM+G model was the best fit for the 16S rRNA partition; for the *ND2* gene, however, the HKY+G model was considered the best fit for the first and second codon partitions, whereas the J2+G model was selected as the best fit for the third codon partition. Mean uncorrected genetic distances (*P*-distances) between sequences were determined with MEGA 7.0 (Kumar et al., 2016).

The matrilineal genealogy was inferred using Bayesian inference (BI) and maximum likelihood (ML) algorithms. BI analyses were conducted in MrBayes v3.1.2 (Huelsenbeck & Ronquist, 2001; Ronquist & Huelsenbeck, 2003). Metropolis-coupled Markov chain Monte Carlo (MCMCMC) analyses were run with one cold chain and three heated chains for twenty million generations and sampled every 2 000 generations. Five independent MCMCMC runs were performed and 1 000 trees were discarded as burn-in. We checked the convergence of the runs and that the effective sample sizes (ESS) were all above 200 by exploring the likelihood plots using TRACER v1.6 (Rambaut et al., 2014). Confidence in tree topology was tested by posterior probability (PP) for the BI trees (Huelsenbeck & Ronquist, 2001). Nodes with PP values over 0.95 were *a-priori* regarded as sufficiently resolved, those between 0.95 and 0.90 were regarded as tendencies, and values below 0.90 were considered to be not supported.

We conducted ML analyses using the RAxML web server (http://embnet.vital-it.ch/raxml-bb/; Stamatakis et al., 2008) and searched ML trees using the gamma model of rate heterogeneity option. Confidence in node topology was tested by non-parametric bootstrapping with 1 000 replicates (ML BS, see Felsenstein, 1985). We *a-priori* regarded tree nodes with bootstrap (ML BS) values of 70% or greater and BI PP values over 0.95 as sufficiently resolved; ML BS values between 70% and 50% (BI PP between 0.95 and 0.90) were treated as tendencies, and nodes with ML BS values below 50% (BI PP below 0.90) were regarded as unresolved (Felsenstein, 2004; Huelsenbeck & Hillis, 1993).

## RESULTS

### Phylogenetic analyses

**Sequences and statistics:** The final alignment of the *ND2* gene contained 1 157 aligned characters, including 712 conserved sites and 445 variable sites, of which 405 were parsimony-informative. The transition-transversion bias (R) was estimated to be 4.56 (all data for ingroup only). Nucleotide frequencies were 37.5% (A), 23.7% (T), 28.3% (C), and 10.5% (G). The final alignment of the 16S rRNA gene contained 508 aligned characters, including 424 conserved sites and 82 variable sites, of which 69 were parsimony-informative. The transition-transversion bias (R) was estimated to be 5.84 (all data for ingroup only). Nucleotide frequencies were 36.8% (A), 24.9% (T), 20.3% (C), and 18.0% (G).

**Position of *Tylototriton* sp. in matrilineal genealogy:** BI and ML phylogenetic analyses resulted in essentially similar topologies (Figure 2). In general, the topology of the mtDNA-based matrilineal genealogy was consistent with the phylogeny of *Tylototriton* presented by Wang et al. (2018), suggesting that the genus is divided into five clades (1–5) grouped into two major reciprocally monophyletic groups (Figure 2):

Specimen numbers (1–115), corresponding voucher specimen information, and GenBank accession Nos. are given in Table 1. Color denotes five major clades revealed within genus *Tylototriton* (clades 1–5). Information on type species of subgeneric-level taxa *Tylototriton* s. str., *Qiantriton*, *Liangshantriton*, and *Yaotriton* is provided. Number at tree nodes corresponds to BI PP/ML BS support values, respectively. Photo by Nikolay A. Poyarkov.

(1) The first group joined two clades with sister relationships: clade 1 (including *T. verrucosus* (type species of *Tylototriton* s. str. Anderson, 1871), *T. anguliceps*, *T. himalayanus*, *T. kweichowensis* (type species of *Qiantriton* Fei, Ye & Jiang, 2012), *T. ngarsuensis*, *T. podichthys*, *T. pulcherrimus*, *T. shanjing*, *T. shanorum*, *T. uyenoi*, *T. yangi*,and *Tylototriton* sp. 1 from the western part of the Kachin and Sagaing States of Myanmar and the newly discovered population of *Tylototriton* **sp. nov.** from the Indawgyi Lake area in the southern part of Kachin State) and clade 2 (including *T. pseudoverrucosus* and *T. taliangensis*, the latter being the type species of *Liangshantriton* Fei, Ye & Jiang, 2012).

(2) The second group joined clade 3 (including *T. broadoridgus*, *T. dabienicus*, *T. liuyangensis*, *T. lizhenchangi*,and *T. wenxianensis*), clade 4 (including *T. panhai* and *T. vietnamensis*), and clade 5 (including *T. asperrimus* (type species of *Yaotriton* Dubois & Raffaëlli, 2009), *T. hainanensis*, *T. notialis*, and *T. ziegleri*); the topological relationships between these three clades are essentially unresolved.

In accordance with the results ofWang et al. (2018), our analysis indicated deep phylogenetic structuring and paraphyly of *T. asperrimus* (consisting of two non-monophyletic lineages), *T. wenxianensis* (consisting of three lineages, not forming a monophyly), and *T. dabienicus* (consisting of two non-monophyletic lineages), suggesting that taxonomy of this group is incomplete and further taxonomic and phylogenetic research is required.

The newly discovered population of *Tylototriton* **sp. nov.** from the Indawgyi Lake area belongs to clade 1 (Figure 2), which occurs in western and northern Indochina, Himalaya, and Yunnan Province of China (Figure 1), and is grouped with *T. himalayanus* from Nepal, though not with significant node support (0.85/57, hereafter given for BI PP/ML BS, respectively). *Tylototriton* **sp. nov.** from the Indawgyi Lake area and *T. himalayanus* form a well-supported monophyletic group with *Tylototriton* species from the Shan Plateau in Myanmar (1.0/96), whereas populations of *Tylototriton* sp. 1 from the Sagaing and Kachin states in northern Myanmar, reported by Grismer et al. (2018a), are clustered with other members of *Tylototriton* clade 1 from Yunnan and northern Indochina and belong to the *T. verrucosus* species complex (Figure 2). Our analyses suggest sister relationships of closely related *T. verrucosus* and *T. shanjing* but only with support from BI (1.0/-), suggesting that the taxonomic status of *T. shanjing* might need to be reconsidered. Finally, in our tree, the recently described Myanmar species *T. ngarsuensis* is nested within differentiation of *T. shanorum*, rendering the latter paraphyletic.

**Sequence divergence:** The uncorrected *P*-distances among and within the studied mtDNA fragments for the examined *Tylototriton* species are shown in Table 2 (data for ingroup only). The interspecific uncorrected genetic *P*-distances between the *Tylototriton* sp. from Kachin State of Myanmar and other congeners varied from 5.3% (between *Tylototriton* sp. and its sister species *T. himalayanus*) to 14.6% (between *Tylototriton* sp. and *T. lizhenchangi*) for the *ND2* gene; and from 2.4% (between *Tylototriton* sp. and its sister species *T. himalayanus*) to 5.9% (between *Tylototriton* sp. and *T. liuyangensis* and *T. dabienicus*, lineage 1) for the 16S rRNA gene (Table 2). This degree of pairwise divergence is quite high, notably greater than the genetic divergence observed between many recognized species of *Tylototriton* (see Table 2 and Grismer et al., 2018a; Wang et al., 2018).

**Table 2:** Uncorrected *P*-distance (percentage) between sequences of 1 157 bp *ND2* gene fragment (below diagonal) and 508 bp 16S rRNA gene fragment (above diagonal) of *Tylototriton* species included in phylogenetic analyses

**Species**		**1**	**2**	**3**	**4**	**5**	**6**	**7**	**8**	**9**	**10**	**11**	**12**	**13**	**14**	**15**	**16**	**17**	**18**	**19**	**20**	**21**	**22**	**23**	**24**	**25**	**26**	**27**	**28**	**29**	**30**	**31**
**1**	*T. kweichowensis*	**–**	–	0.0	3.3	2.0	4.4	–	–	2.8	2.4	2.7	2.5	4.0	2.4	2.8	3.8	0.0	3.4	3.8	–	4.4	4.2	4.0	3.8	3.8	4.7	3.7	3.4	3.4	–	–
**2**	*T. shanorum* 1	5.9	**–**	–	–	–	–	–	–	–	–	**–**	–	–	–	–	–	–	–	–	–	–	–	–	–	–	–	–	–	–	–	–
**3**	*T. shanorum* 2	5.6	0.6	**–**	–	–	–	–	–	–	–	–	**–**	–	–	–	–	–	–	–	–	–	–	–	–	–	–	–	–	–	–	–
**4**	*T. himalayanus*	5.2	5.1	4.9	**–**	2.3	4.8	–	–	3.7	3.7	3.5	2.4	3.8	4.3	4.5	4.1	3.3	4.5	4.5	–	4.7	5.2	4.3	4.3	4.3	4.5	3.9	4.5	4.2	–	–
**5**	*T. yangi*	6.1	6.6	6.4	6.5	**–**	2.6	–	–	1.6	2.0	1.8	3.2	3.5	2.4	2.8	3.8	2.0	2.8	3.2	–	3.6	3.2	3.2	2.8	2.8	3.3	2.3	2.8	2.5	–	–
**6**	*T. uyenoi*	8.1	9.2	9.1	8.5	7.5	**–**	–	–	3.1	3.1	3.2	5.2	6.2	4.4	4.8	4.8	4.4	4.8	4.8	–	5.1	5.3	5.5	5.1	4.6	5.6	5.0	5.3	4.8	–	–
**7**	*T. anguliceps*	6.3	7.0	6.8	7.0	4.5	7.6	**–**	–	–	–	–	–	–	–	–	–	–	–	–	–	–	–	–	–	–	–	–	–	–	–	–
**8**	*T. podichthys*	5.8	7.2	6.9	6.7	5.0	8.7	5.4	**–**	–	–	–	–	–	–	–	–	–	–	–	–	–	–	–	–	–	–	–	–	–	–	–
**9**	*T. pulcherrimus*	5.3	6.4	6.1	6.3	3.8	6.8	4.2	3.3	**–**	1.6	1.0	3.9	4.8	2.2	2.6	4.2	2.8	3.4	3.6	–	4.0	3.8	3.6	3.6	3.6	4.5	3.5	3.8	3.3	–	–
**10**	*T. verrucosus*	5.6	6.2	6.0	6.4	3.9	7.2	4.6	3.6	2.1	**–**	0.7	3.9	5.3	2.0	2.6	4.4	2.4	3.6	3.8	–	4.2	4.2	3.8	4.2	3.8	4.7	3.7	4.0	3.6	–	–
**11**	*T. shanjing*	5.9	6.6	6.4	6.2	4.5	7.2	4.8	4.0	2.6	1.1	**–**	3.7	5.2	2.3	2.5	4.3	2.7	3.5	3.7	–	4.1	4.0	3.7	4.0	3.7	4.5	3.6	3.9	3.5	–	–
**12**	*T. kachinorum * **sp. nov.**	6.5	7.6	7.1	5.3	8.2	11.6	8.3	8.0	7.7	7.3	7.9	**–**	5.2	4.1	4.3	4.7	2.5	5.2	5.2	–	5.5	5.9	5.0	5.0	5.0	5.9	5.0	5.2	4.9	–	–
**13**	*T. panhai*	10.7	12.2	12.1	11.9	12.7	14.3	13.3	12.4	11.8	12.7	13.0	13.3	**–**	5.1	4.8	4.4	4.0	4.4	4.8	–	4.7	4.7	4.2	4.2	4.2	4.7	3.6	4.0	3.8	–	–
**14**	*T. taliangensis*	6.3	8.2	8.0	7.6	7.9	9.7	8.8	7.9	7.3	7.4	7.5	7.3	10.5	**–**	1.2	4.2	2.4	3.2	3.6	–	4.0	3.8	3.2	4.0	3.6	4.5	3.5	3.6	3.2	–	–
**15**	*T. pseudoverrucosus*	5.7	7.9	7.7	7.1	7.4	9.7	8.6	7.4	6.6	7.4	7.7	7.1	10.3	2.7	**–**	4.0	2.8	3.2	3.6	–	3.8	3.8	3.8	4.4	4.0	4.9	3.9	3.2	3.6	–	–
**16**	*T. vietnamensis*	11.8	12.5	12.2	12.7	12.2	15.0	13.4	12.6	12.0	12.1	12.5	13.1	11.0	11.2	11.7	**–**	3.8	4.0	4.0	–	3.8	4.5	4.2	4.2	3.8	4.3	4.2	4.0	4.0	–	–
**17**	*T. ziegleri*	9.4	11.1	11.1	11.2	10.2	13.4	11.2	10.9	10.6	11.3	11.4	11.8	10.3	9.7	9.3	11.3	**–**	3.4	3.8	–	4.4	4.2	4.0	3.8	3.8	4.7	3.7	3.4	3.4	–	–
**18**	*T. hainanensis*	8.5	9.9	9.8	9.9	9.6	12.6	10.3	9.9	9.5	10.2	10.3	11.9	9.6	8.6	8.4	10.8	4.4	**–**	0.8	–	1.4	3.1	2.8	3.4	3.0	3.9	2.9	2.6	2.7	–	–
**19**	*T. asperrimus* 2	9.3	10.2	10.0	10.0	9.3	13.2	10.0	10.1	10.0	10.5	10.7	13.0	11.4	9.3	9.1	11.6	5.6	3.6	**–**	–	1.4	3.5	2.8	3.4	3.0	3.9	3.0	3.0	3.0	–	–
**20**	*T. notialis*	9.7	10.7	10.7	10.6	10.3	12.7	10.9	10.7	10.2	10.9	11.0	12.4	10.5	9.2	8.9	11.6	4.9	4.7	5.9	**–**	–	–	–	–	–	–	–	–	–	–	–
**21**	*T. asperrimus* 1	10.0	11.2	11.2	11.4	10.2	13.4	11.4	10.8	10.9	11.4	11.5	13.1	11.4	9.4	9.5	10.9	4.5	4.9	5.7	4.8	**–**	3.9	3.0	3.9	3.4	4.3	3.4	3.7	3.4	–	–
**22**	*T. liuyangensis*	9.4	10.3	10.3	10.1	9.5	12.7	10.4	10.5	9.9	10.3	10.7	12.3	9.5	9.1	9.0	10.9	8.5	7.8	8.5	8.6	8.6	**–**	2.4	1.9	1.5	2.4	2.0	1.3	1.7	–	–
**23**	*T. lizhenchangi*	10.7	11.9	11.8	11.5	11.1	13.2	12.6	11.9	10.9	11.5	11.7	14.6	9.8	9.5	9.8	11.7	10.0	8.7	9.6	9.7	10.4	7.1	**–**	2.0	1.6	2.5	2.0	1.8	1.6	–	–
**24**	*T. dabienicus* 2	9.7	10.8	10.8	10.9	10.1	12.7	11.1	9.9	9.9	10.6	10.5	10.9	9.7	9.0	9.4	10.8	8.3	7.9	8.8	8.2	8.1	7.3	8.3	**–**	0.8	1.3	0.9	1.2	1.3	–	–
**25**	*T. broadoridgus*	9.1	10.3	10.2	10.5	9.9	12.3	11.0	9.9	9.7	10.1	10.2	11.2	9.5	9.1	9.0	10.7	8.0	7.5	8.8	7.9	8.0	7.0	8.1	3.4	**–**	0.9	0.9	0.8	1.1	–	–
**26**	*T. dabienicus* 1	10.2	11.0	11.1	11.2	11.0	13.0	12.2	10.9	10.5	10.9	11.1	11.6	9.7	9.2	9.6	10.9	9.1	8.9	9.6	8.9	9.0	7.4	8.8	3.9	3.4	**–**	1.0	1.7	1.9	–	–
**27**	*T. wenxianensis* 3	9.1	10.2	10.2	10.3	9.9	12.0	11.2	10.0	9.6	10.3	10.4	10.8	9.2	8.8	8.9	10.7	7.8	7.5	8.5	7.8	8.2	7.0	7.8	3.2	2.2	2.5	**–**	1.2	1.3	–	–
**28**	*T. wenxianensis* 2	9.5	10.3	10.4	10.7	9.7	12.5	11.0	10.1	9.7	10.0	10.2	11.7	10.3	9.0	9.2	11.0	8.6	8.2	9.0	8.2	8.5	7.2	8.0	3.3	2.8	3.6	2.9	**–**	1.1	–	–
**29**	*T. wenxianensis* 1	9.9	11.0	11.1	10.9	10.0	12.7	11.4	10.0	9.6	10.1	10.3	11.9	10.5	9.1	9.4	10.7	9.3	8.9	9.5	8.9	9.0	7.3	8.3	4.4	4.1	4.6	4.0	4.0	**–**	–	–
**30**	*T. ngarsuensis*	6.6	1.8	1.5	6.2	6.9	9.3	7.5	7.6	6.8	6.7	7.0	8.0	12.5	9.1	8.6	13.1	11.9	10.6	10.5	11.8	11.9	11.3	12.6	11.7	11.3	12.5	11.5	11.6	11.9	**–**	–
**31**	*Tylototriton* sp. 1	5.7	6.7	6.4	6.7	4.2	7.7	4.7	3.5	2.8	2.5	3.2	6.6	12.5	7.5	7.3	12.1	10.8	10.1	10.1	10.8	10.9	10.2	11.4	10.2	10.0	10.5	10.2	10.0	10.1	6.8	**–**

### Taxonomy

Our mtDNA genealogy analyses based on the *ND2* and 16S rRNA genes indicated that the newly discovered population of *Tylototriton* **sp. nov.** from the Indawgyi Lake area belongs to clade 1 of the subgenus *Tylototriton* s. str. and is clustered with two other species of the genus known from Myanmar, *T. ngarsuensis* and *T. shanorum*, and with *T. himalayanus* from Nepal (Figure 2). The lineage of *Tylototriton* **sp. nov.** from Indawgyi Lake is clearly distinct and notably divergent from all other congeners with the uncorrected genetic distance for interspecific comparisons exceeding *P*=5.3% in the *ND2* gene and *P*=2.4% in the 16S rRNA gene. The observed differences in mtDNA sequences are congruent with evidence from diagnostic morphological characters (see “Comparisons”). These results support our hypothesis that the newly discovered population of *Tylototriton* **sp. nov.** from Indawgyi Lake represents a previously unknown species, which we describe herein.

*Tylototriton kachinorum* **sp. nov.**

**Figure 3:**
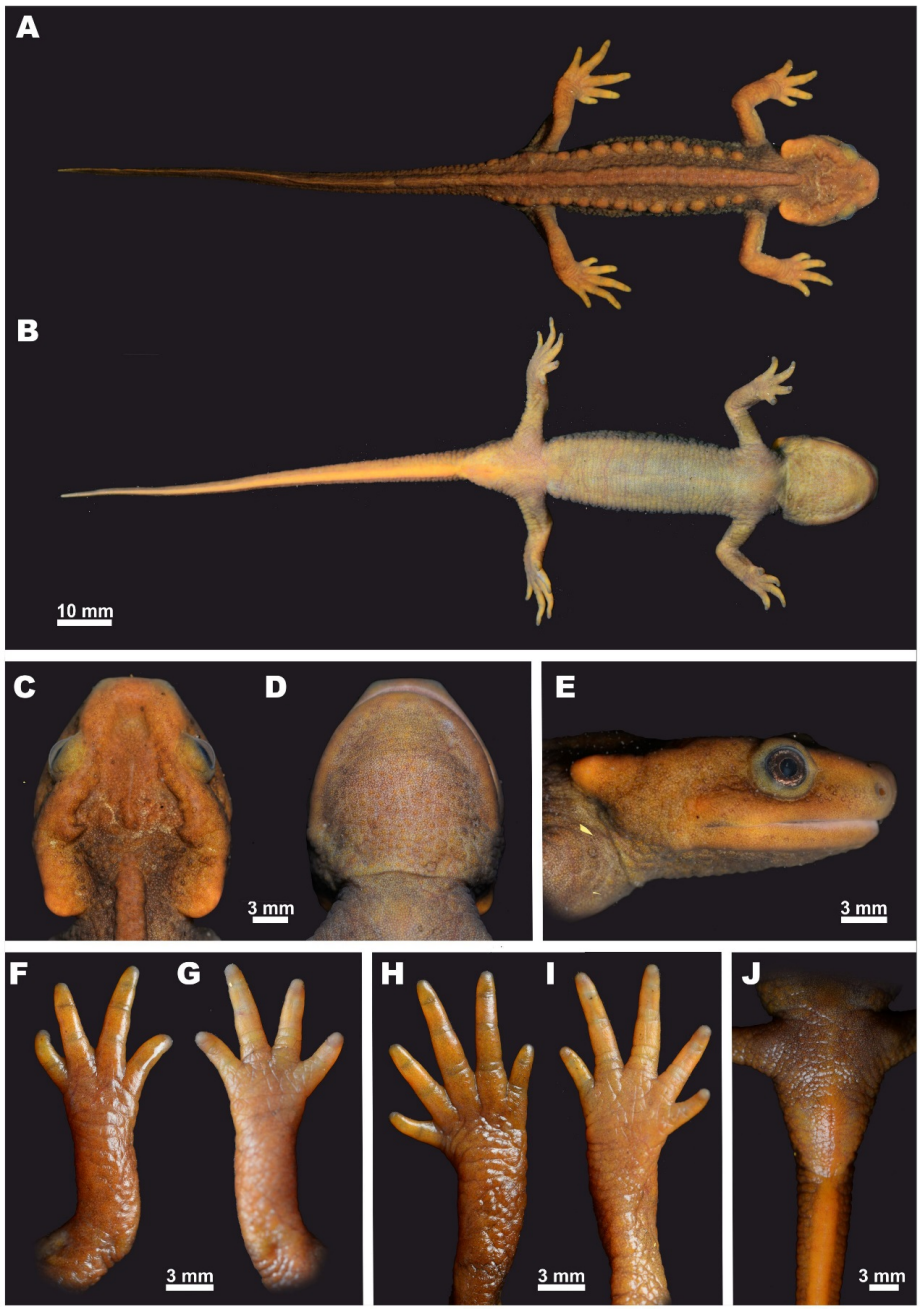
Holotype of *Tylototriton kachinorum *sp. nov. (ZMMU A5953, male) in life

**Figure 4:**
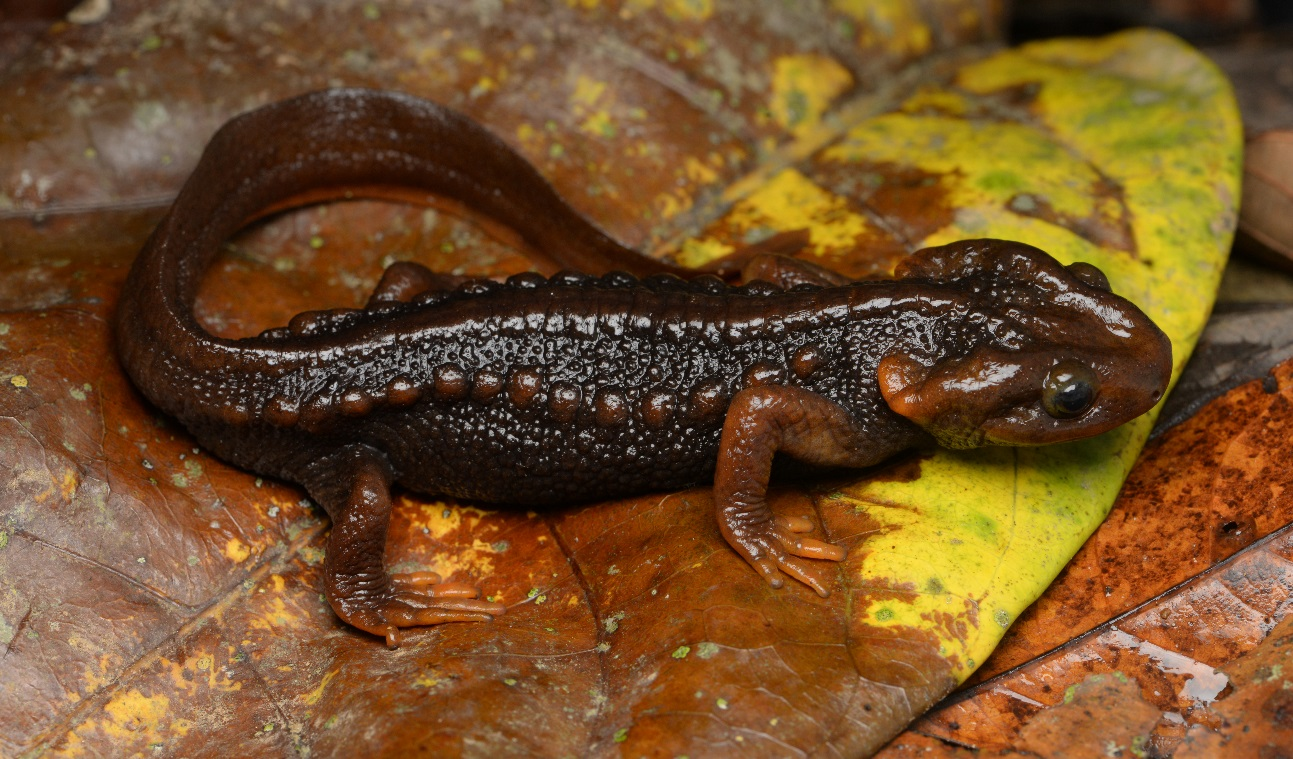
Holotype of *Tylototriton kachinorum *sp. nov. (ZMMU A5953, male) *in situ* (Photo by Nikolay A. Poyarkov and Than Zaw)

**Figure 5:**
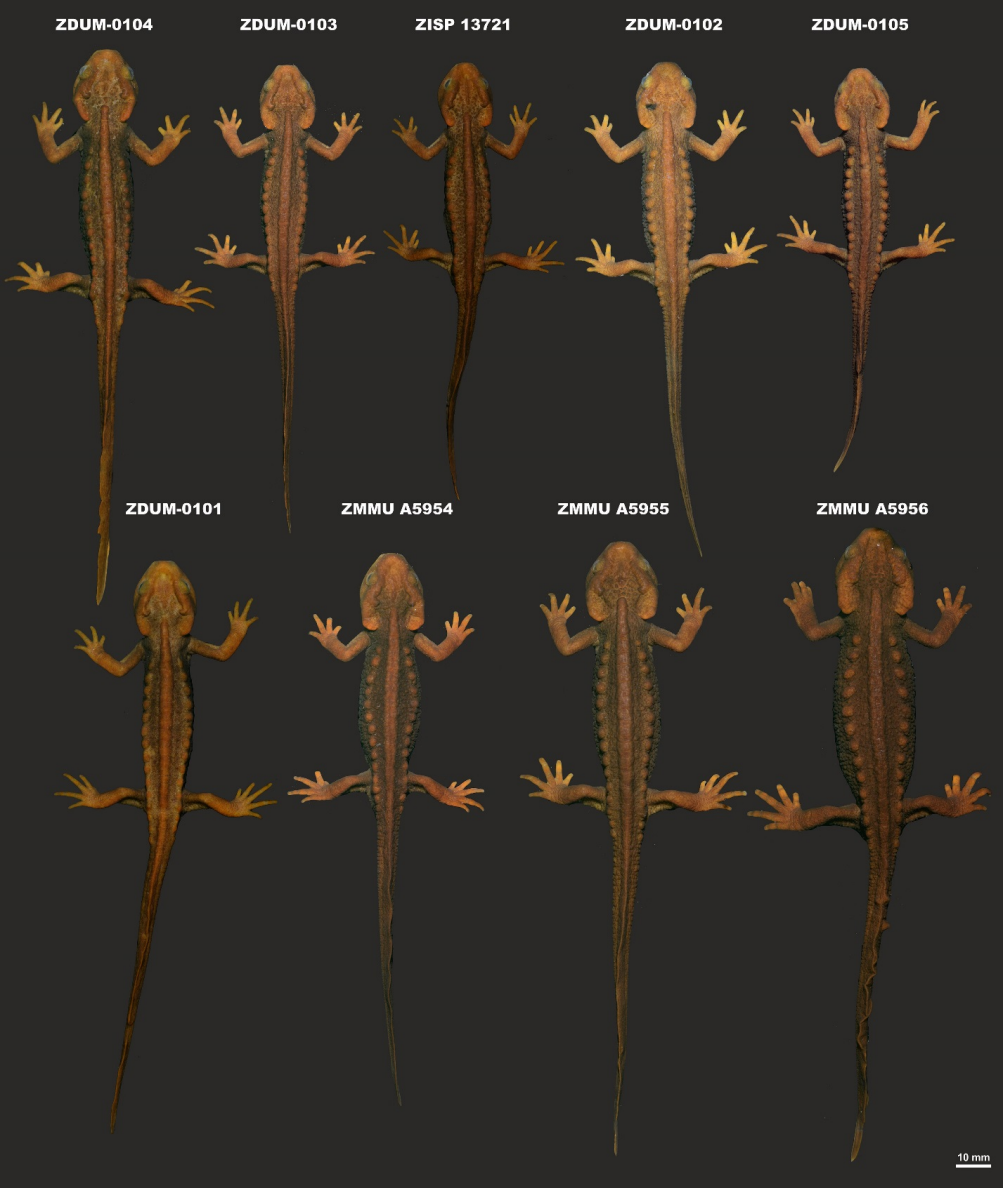
Variation of dorsal coloration in paratypes of *Tylototriton kachinorum *sp. nov.

**Figure 6:**
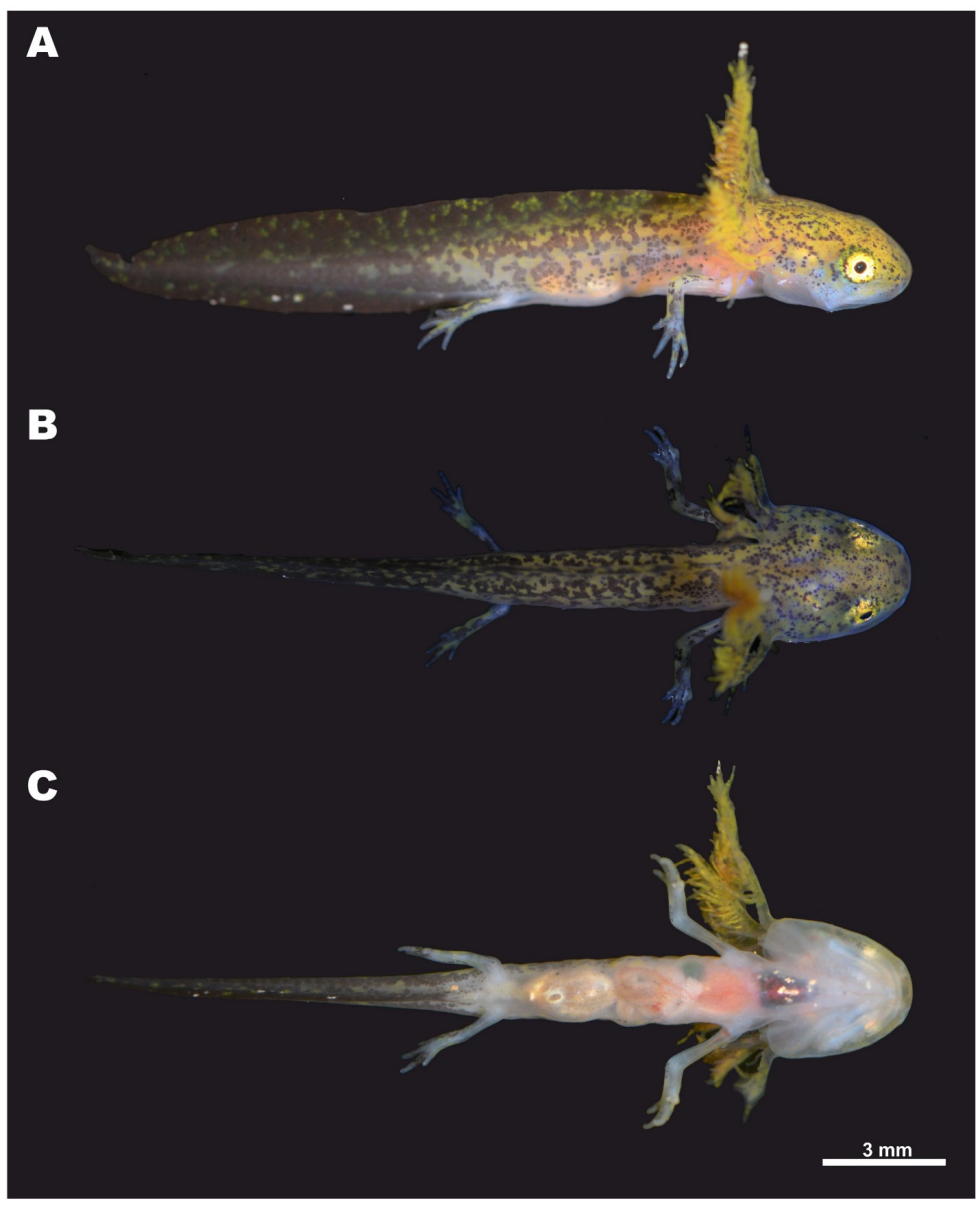
Lateral (A), dorsal (B), and ventral (C) views of larval specimen (ZMMU A5957; Grosse (2013) stage 40) of *Tylototriton kachinorum *sp. nov. in life

Tables 3–4; Figures 3–6.

**Holotype:** ZMMU A5953 (field number NAP-08318), adult male from a swamp in a forest clearing surrounded by montane evergreen tropical forest, Ingyin Taung Mountain, Indawgyi Lake area, Mohnyin Township, Kachin State, Myanmar (approximate coordinates N25.09°, E96.28°; elevation 1 000 m a.s.l.), collected on July 18, 2018 at 2100 h by Than Zaw, Paw Lay, Parinya Pawangkhanant, Vladislav A. Gorin, and Nikolay A. Poyarkov.

**Paratypes:** ZMMU A5954 (field number NAP-08320), ZISP 13721 (field number NAP-08324), ZDUM-0101–0105 (field numbers NAP-08325, NAP-08322, NAP-08319, NAP-08326 and NAP-08317, respectively), seven adult males from the same locality and with the same collection information as the holotype; and ZMMU A5955–A5956 (field numbers NAP-08323 and NAP-08321, respectively), two adult females from the same locality and with the same collection information as the holotype.

**Referred specimens:** ZMMU A5957 (field number NAP-08305), a larva (Grosse (2013) stage 40) from the same locality and with the same collection information as the holotype.

**Diagnosis:** The new species is assigned to the genus *Tylototriton* based on molecular data and by the following combination of morphological attributes: (1) presence of dorsal granules, (2) dorsolateral bony ridges on head, (3) presence of dorsolateral series of knob-like warts (rib nodules); and (4) absence of quadrate spine ( Figure 2). *Tylototriton kachinorum* **sp. nov.** is distinguished from all other congeners by a combination of the following morphological attributes: (1) medium body size, adult SVL 62.3–74.1 mm in males, 72.5–84.8 mm in females; (2) tail thin and long, longer than body in both sexes, lacking lateral grooves; (3) skin rough with fine granules; (4) snout truncate in dorsal view; (5) supratemporal bony ridges on head wide, protruding, beginning at anterior corner of orbit; (6) sagittal ridge on head very weak, almost indistinct; (7) limbs long and thin, tips of forelimb and hindlimb broadly overlapping when adpressed along body; (8) vertebral ridge distinct, wide, non-segmented; (9) rib nodules weakly distinct, 13–14 along each side of body; (10) background coloration brown to dark-brown; (11) labial regions, parotoids, rib nodules, whole limbs, vent, ventral tail ridge with dull orange-brown to yellowish-brown markings.

The new species is also markedly distinct from all congeners for which comparable sequences are available of *ND2* (*P*≥5.3%) and 16S rRNA (*P*≥2.4%) mitochondrial DNA genes.

**Description of holotype:** A medium-sized specimen in a good state of preservation (Figures 3–4).

**Head:** Head longer than wide (HW/HL ratio 89.3%) (Figure 3C), head wider than body; angularly hexagonal in shape in dorsal view, slightly depressed, gently sloping in profile (Figure 3E); snout comparatively long, three times longer than eye (UEW/SL ratio 33.9%), sharply truncate in dorsal view (Figure 3C), slightly rounded in lateral view (Figure 3E), slightly projecting beyond lower jaw; nostrils on anterior margin of snout located notably closer to snout tip than to eye (NSD/ON ratio 58.8%), facing anterolaterally, not visible from dorsal view; labial folds absent; tongue oval, attached to anterior floor of mouth but free posteriorly and laterally; vomerine teeth arranged in inverted V-shaped almost straight series, converging and narrow anteriorly, gradually widening posteriorly, notably longer than wide (VTW/VTL ratio 77.7%), anteriorly reaching beyond level of choanae but not in contact with them, vomerine teeth 95 (47/48 in right and left branches, respectively), upper jaw teeth 93, and lower jaw teeth 110; parotoids distinct, comparatively large, crescent-shaped, notably projecting posteriorly (Figure 3E); dorsolateral supratemporal bony ridges on head wide, notably protruding, from anterior corner of orbit to anterior end of parotoid, forming medially recurved projection on its posterior end (Figure 3C); sagittal bony ridge on head very weak, almost indistinct (Figure 3C); gular fold present (Figure 3D).

A: Dorsal view; B: Ventral view; C: Head, dorsal view; D: Head, ventral view; E: Head, lateral view; F: Opisthenar view of right hand; G: Volar view of right hand; H: Opisthenar view of right foot; I: Plantar view of right foot; J: Ventral view of cloacal area. Photos by Nikolay A. Poyarkov.

**Body:** Body habitus comparatively slender (Figure 3A); costal folds absent; vertebral middorsal ridge wide, non-segmented, running from occiput region to anterior one fifth of tail length, separated from sagittal head ridge on head with wide gap (Figure 3C); rib nodules weakly distinct, small, forming knob-like glandular warts, arranged in two longitudinal lines on dorsolateral surfaces of dorsum, 14 on both sides of body from area posterior to axilla to level of posterior vent margin (base of tail) (Figure 3A); rib nodules almost of same size, rounded, those in posterior third of dorsum slightly oval-shaped, those on sacral area notably elongated, decreasing in size posteriorly on sacrum and tail basis. **Limbs:** Limbs comparatively long, slender (Figure 3A); forelimbs slightly shorter than hindlimbs; relative length of forelimb FLL/SVL ratio 26.2%, relative length of hindlimb ratio 28.0%; fore- and hindlimbs largely overlapping when adpressed towards each other along sides of body; fingers and toes well developed (Figure 3F–I), free of webbing; fingers four, comparative finger lengths: 1FL<4FL<2FL<3FL; toes five, comparative toe lengths: 1TL<5TL<2TL<3TL<4TL. **Tail:** Tail very long, notably exceeding body length (TAL/SVL ratio 114.9%); tail laterally compressed along entire length, tapering posteriorly, lateral grooves on tail absent; dorsal tail fin starting at anterior one fifth of tail length, more distinct posteriorly, with maximal tail height at posterior two thirds of tail length, dorsal tail fin slightly serrated; ventral tail fin smooth; tail tip pointed. **Skin texture and skin glands:** Skin rough, small granules present on dorsal surfaces of head and dorsum (Figure 3A,C), lateral sides of body and tail; on ventral surface granules become smaller, arranged in transverse striations (Figure 3B); small, sparse granules regularly arranged on throat (Figure 3D); head ridges with rough surface; skin on volar and plantar surfaces of hands and feet with tiny grooves forming reticulated pattern; metacarpal or metatarsal tubercles absent. Cloacal region notably swollen, vent as longitudinal slit (Figure 3J), vent edges with numerous transverse folds.

**Color of holotype in life: **Dorsal ground color of dorsal surfaces of head and trunk dark brown(Figure 4); dorsal surfaces of limbs and lateral surfaces of tail light yellowish-brown (Figure 3A); iris brown with tiny bronze speckles along outer margins (Figure 3E); gular region, belly, and ventral surfaces of limbs light yellowish-gray (Figure 3B); anterior parts of head and parotoids light orange-brown; rib nodules and vertebral ridge yellowish-brown to orange-brown, barely discernable from dark brown trunk coloration; upper and lower lips, posteriormost corners of parotoids, palms, and soles light-orange to yellowish.

**Color of holotype in preservative:** After preservation in ethanol for six months, coloration pattern of holotype resembles that observed in life, however yellowish and orange tints faded to light brownish-gray.

**Measurements and counts of holotype:** Morphometric characters (all in mm): SVL 67.2; RHL 18.1; RHW 16.2; RMXHW 16.6; RIND 5.4; RAGD 34.1; RTRL 50.4; TAL 77.3; RVL 6.3; RFLL 26.2; RHLL 28.0; RVTW 6.0; RVTL 7.7; RLJL 13.5; RSL 6.8; RIOD 7.2; RUEW 2.3; RUEL 4.4; ROL 3.3; RBTAW 9.0; RMTAW 3.2; RMXTAH 10.8; RMTAH 7.0; RON 4.6; ICD 9.6; CW 11.5; NSD 2.7; 1FL 2.9; 2FL 4.8; 3FL 5.5; 4FL 3.0; 1TL 2.5; 2TL 5.4; 3TL 7.0; 4TL 7.7; 5TL 3.6. Meristic characters: UJTN 93; LJTN 110; VTN 47/48 (right/left).

**Variation:** All individuals in the type series were generally similar in morphology and agreed with the holotype description in body proportions and coloration; variation of morphometric characters within the type series is shown in Table 3. The variation of dorsal coloration in seven male and two female paratypes in life is presented in Figure 5. In general, males had more robust and slender bodies than females. The males were notably smaller in body size (SVL 62.3–74.1 mm, mean 68.6±2.9 mm) than the two females (SVL 72.5–84.8 mm) (Table 3). Body of the largest female (ZMMU A5956) was notably swollen and was wider than head width (Figure 5). Male paratypes ZDUM-0105 and ZISP 13721 had notably shorter tails than other type specimens (Table 4) due to regeneration of tail tip after damage (Figure 5). Coloration within the type series slightly varied, from specimens lighter than the holotype, which appeared light-brown to yellowish-orange (ZDUM-0102), to darker specimens, which appeared dark-brown with duller orange-brown light markings (male ZISP 13721 and female ZMMU A5956) (Figure 5).

Scale bar: 10 mm. Photos by Nikolay A. Poyarkov.

**Table:** Measurements of type series of *Tylototriton kachinorum *sp. nov. (all in mm)

Specimen	ZMMU A5953	ZMMU A5954	ZDUM-0101	ZDUM-0102	ZDUM-0103	ZDUM-0104	ZDUM-0105*	ZISP 13721*	**M**		ZMMU A5955	ZMMU A5956	**F**
Type status	Holotype	Paratype	Paratype	Paratype	Paratype	Paratype	Paratype	Paratype			Paratype	Paratype	
Sex	M	M	M	M	M	M	M	M	**Mean±*SD***	**(Min–Max)**	F	F	**Mean±*SD***
SVL	67.2	74.1	74.0	68.3	66.9	69.3	62.3	66.3	**68.6±2.9**	**(62.3–74.1)**	72.5	84.8	**78.7±6.2**
HL	18.1	20.7	20.7	18.6	19.6	19.7	16.9	17.3	**18.9±1.2**	**(16.9–20.7)**	18.1	20.3	**19.2±1.1**
HW	16.2	19.2	18.5	17.0	16.7	17.7	15.8	16.4	**17.2±1.0**	**(15.8–19.2)**	18.4	21.4	**19.9±1.5**
MXHW	16.6	19.6	18.6	17.5	17.6	18.4	16.6	16.7	**17.7±0.9**	**(16.6–19.6)**	19.4	23.0	**21.2±1.8**
IND	5.4	6.4	5.7	5.4	5.2	5.6	4.2	4.8	**5.3±0.4**	**(4.2–6.4)**	5.8	6.8	**6.3.±0.5**
AGD	34.1	36.6	36.6	34.0	33.1	33.9	30.8	33.2	**34.0±1.3**	**(30.8–36.6)**	40.5	48.2	**44.4±3.9**
TRL	50.4	56.2	55.1	51.3	50.4	52.9	47.1	49.4	**51.6±2.3**	**(47.1–56.2)**	58.4	69.0	**63.7±5.3**
TAL	77.3	89.6	96.3	79.2	79.2	85.2	57.0	67.8	**78.9±8.7**	**(57.0–96.3)**	82.7	85.2	**84.0±1.3**
VL	6.3	7.4	9.7	6.8	6.2	7.4	5.7	6.5	**7.0±0.9**	**(5.7–9.7)**	4.9	4.8	**4.9±0.1**
FLL	26.2	29.3	29.1	26.5	26.1	28.2	24.1	26.4	**27.0±1.4**	**(24.1–29.3)**	26.1	29.9	**28.0±1.9**
HLL	28.0	31.0	31.6	28.0	27.3	29.7	26.6	27.5	**28.7±1.5**	**(26.6–31.6)**	29.0	32.2	**30.6±1.6**
VTW	6.0	6.7	6.6	6.2	6.1	6.3	5.6	5.9	**6.2±0.3**	**(5.6–6.7)**	6.4	7.4	**6.9±0.5**
VTL	7.7	9.1	9.4	8.8	8.9	9.2	8.4	8.8	**8.8±0.4**	**(7.7–9.4)**	8.4	9.7	**9.1±0.6**
LJL	13.5	16.0	14.1	13.9	13.7	14.4	12.3	13.4	**13.9±0.7**	**(12.3–16.0)**	14.5	16.9	**15.7±1.2**
SL	6.8	7.6	8.1	7.3	7.0	7.5	6.2	6.5	**7.1±0.5**	**(6.2–8.1)**	7.3	8.7	**8.0±0.7**
IOD	7.2	8.4	8.1	7.3	8.0	7.9	7.2	7.3	**7.7±0.4**	**(7.2–8.4)**	7.7	8.7	**8.2±0.5**
UEW	2.3	2.5	2.6	2.4	2.4	2.4	2.2	2.2	**2.4±0.1**	**(2.2–2.6)**	2.3	2.8	**2.6±0.2**
UEL	4.4	5.0	4.9	4.6	4.4	4.6	4.1	4.4	**4.5±0.2**	**(4.1–5.0)**	4.3	5.3	**4.8±0.5**
OL	3.3	3.6	3.6	3.3	3.2	3.4	3.1	3.2	**3.3±0.1**	**(3.1–3.6)**	3.6	4.2	**3.9±0.3**
BTAW	9.0	9.7	9.5	10.0	9.7	9.6	8.8	9.6	**9.5±0.3**	**(8.8–10.0)**	8.5	9.8	**9.2±0.7**
MTAW	3.2	3.3	3.9	3.4	3.6	3.7	3.3	3.6	**3.5±0.2**	**(3.2–3.9)**	3.8	4.2	**4.0±0.2**
MXTAH	10.8	11.8	10.8	10.7	10.1	10.2	9.0	10.9	**10.5±0.6**	**(9.0–11.8)**	11.6	11.9	**11.7±0.2**
MTAH	7.0	9.0	7.3	7.5	7.8	7.7	7.7	8.4	**7.8±0.5**	**(7.0–9.0)**	9.3	9.7	**9.5±0.2**
ON	4.6	5.1	5.1	4.7	4.7	4.7	4.2	4.7	**4.7±0.2**	**(4.2–5.1)**	5.0	5.2	**5.1±0.1**

Character abbreviations: SVL: Snout-vent length; HL: Head length; HW: Head width; MXHW: Maximum head width; IND: Internarial distance; AGD: Axilla-groin distance; TRL: Trunk length; TAL: Tail length; VL: Vent length; FLL: Forelimb length; HLL: Hindlimb length; VTW: Vomerine tooth series width; VTL: Vomerine tooth series length; LJL: Lower jaw length; SL: Snout length; IOD: Interorbital distance; UEW: Upper eyelid width; UEL: Upper eyelid length; OL: Orbit length; BTAW: Basal tail width; MTAW: Tail width at mid-level of tail; MXTAH: Maximum tail height; MTAH: Tail height at mid-level of tail; ON: Orbitonarial distance. M: Male. F: Female. For other abbreviations see “Materials and Methods”. Asterisk (*) denotes damaged and regenerated tail.

**Table 4:** Morphological comparison between *Tylototriton* species found in Myanmar and adjacent territories

**Species**	*Tylototriton kachinorum * **sp. nov.**			*T. shanorum*				*T. ngarsuensis*			
	M		F	M		F		M			F
**Character**	8		2	1		2		2			1
**SVL**	68.6±2.9		72.5–84.8	76.0		76.5–87.9		74.9–76.4			102.3
**RHL**	27.6		23.9–24.9	22.4		24.3–25.7		24.0–26.0			21.5
**RHW**	25.1		25.3–25.4	25.4		24.8–26.3		24.5–28.2			26.6
**RIND**	7.7		8.0	6.7		6.0–6.3		8.0–8.8			7.7
**RAGD**	49.6		55.9–56.8	49.6		49.4–51.9		48.2–49.9			51.2
**RTRL**	75.3		80.5–81.4	77.6		74.3–75.7		74.2–75.5			77.5
**RTAL**	120.5		100.5–114.0	111.2		97.0–97.8		98.0–103.5			104.6
**RVL**	10.2		5.6–6.8	9.3		3.4–3.5		10.7–12.3			8.0
**RFLL**	39.3		35.3–36.0	34.7		31.7–32.5		39.7–39.9			35.5
**RHLL**	41.9		37.9–39.9	37.2		35.6–37.0		41.9–47.1			38.7
**RMXHW**	25.8		26.8–27.1	25.9		25.7–27.1		–			–
**RVTW**	9.0		8.7–8.8	6.1		6.1–7.5		–			–
**RVTL**	12.8		11.4–11.6	10.7		11.4–12.0		–			–
**Snout**	Truncate			Blunt to truncate				Rounded to blunt			
**Supratemporal ridge**	Wide, protruding, begins at anterior corner of orbit			Wide, protruding, begins in loreal region				Wide, not protruding, begins posterior to orbit			
**Sagittal ridge**	Very weak, indistinct			Very weak				Very weak, in males			
**Surface of head ridges**	Rough			Rough				Very rough			
**Adpressed limbs**	Overlapping			Overlapping				Overlapping			
**Vertebral ridge**	Wide, non-segmented			Narrow, weakly segmented				Wide, weakly segmented			
**Rib nodules**	Weakly distinct, 13–14			Distinct, 14				Distinct, 15			
**Ground color**	Brown to dark brown			Dark brown to black				Nearly black			
**Color of light markings**	Orange-brown to yellowish-brown			Yellow to reddish brown				Dark yellow			
**Location of light markings**	Parotoids, rib nodules, palms, soles, vent, ventral tail ridge			Head, vertebral ridge, rib nodules, vent, whole limbs and tail				Labial regions, palms and soles, vent, ventral tail ridge			
**Lateral grooves on tail**	Absent			Absent				Absent			
**Species**	*T. himalayanus*			*T. verrucosus*				*T. shanjing*			
	M	F		M	M			F		M	
**Character**	32	13		3	32			13		3	
**SVL**	71.92±6.1	76.06±7.5		70.7±4.7	71.92±6.1			76.06±7.5		70.7±4.7	
**RHL**	24.5	25.9		24.3	24.5			25.9		24.3	
**RHW**	23.0	23.6		23.7	23.0			23.6		23.7	
**RIND**	8.4	8.2		7.0	8.4			8.2		7.0	
**RAGD**	53.6	51.6		50.7	53.6			51.6		50.7	
**RTRL**	79.9	77.1		75.7	79.9			77.1		75.7	
**RTAL**	98.0	100.6		104.9	98.0			100.6		104.9	
**RVL**	11.8	12.5		7.0	11.8			12.5		7.0	
**RFLL**	36.2	36.1		34.3	36.2			36.1		34.3	
**RHLL**	37.5	38.1		37.3	37.5			38.1		37.3	
**RMXHW**	–	–		25.3	–			–		25.3	
**RVTW**	–	–		7.7	–			–		7.7	
**RVTL**	–	–		9.8	–			–		9.8	
**Snout**	Blunt			Truncate				Rounded			
**Supratemporal ridge**	Very wide, protruding			Narrow, steep				Narrow, steep			
**Sagittal ridge**	Weak, glandular			Weak				Short, weak			
**Surface of head ridges**	Very rough			Smooth				Smooth			
**Adpressed limbs**	Overlapping			Overlapping				Overlapping			
**Vertebral ridge**	Non-segmented			Well segmented				Well segmented			
**Rib nodules**	Large, prominent, 16			Weakly distinct, 14–15				Distinct, 14			
**Ground color**	Brown to dark brown			Blackish				Blackish			
**Color of light markings**	Light brown			Orange				Bright to dark orange			
**Location of light markings**	Ventral surface of limbs, vent, ventral tail ridge			Palms, soles, vent and ventral ridge of tail				Head, vertebral ridge, rib nodules, vent, whole limbs and tail			
**Lateral grooves on tail**	Very distinct			Absent				Weak			
**Species**	*T. uyenoi*			*T. anguliceps*				*T. podichthys*			
	M	F		M			F	M	F		
**Character**	9	2		2			5	2	2		
**SVL**	68.1±3.8	69.3–78.3		61.1–62.5			70.6±3.4	56.5–60.2	73.4–78.3		
**RHL**	24.7	25.8–26.9		26.2–29.5			23.4	32.6–34.3	28.1–29.3		
**RHW**	25.0	23.1–24.0		22.7–23.4			22.7	26.4–28.0	24.8–25.9		
**RIND**	7.0	7.0–7.1		6.6–7.4			6.5	8.3–8.7	7.5–7.9		
**RAGD**	48.8	47.9–50.7		47.2–47.8			52.8	48.0–50.7	52.1–53.5		
**RTRL**	75.3	73.1–74.2		70.5–73.8			76.6	65.7–67.4	70.7–71.9		
**RTAL**	115.0	88.0–97.0		97.1–102.3			91.2	80.2–104.8	79.2–81.4		
**RVL**	7.4	1.7–1.9		7.2–7.2			2.5	14.2–14.8	6.3–7.7		
**RFLL**	34.3	30.6–32.7		32.7–34.9			30.2	39.4–40.5	34.0–36.2		
**RHLL**	37.5	37.4–37.4		36.8–37.3			33.3	38.2–40.2	35.8–36.1		
**RMXHW**	25.9	23.6–25.0		23.7–24.1			23.3	–	–		
**RVTW**	7.6	6.6–7.0		6.4–7.8			6.9	–	–		
**RVTL**	10.3	8.8–10.2		9.9–10.8			9.2	–	–		
**Species**	*T. uyenoi*			*T. anguliceps*				*T. podichthys*			
**Snout**	Rounded			Truncate				Rounded			
**Supratemporal ridge**	Narrow, steep			Narrow, very steep				Very wide, protruding			
**Sagittal ridge**	Short, weak			Long, protruding				Weak, glandular			
**Surface of head ridges**	Smooth			Rough				Very rough			
**Adpressed limbs**	Overlapping			Overlapping				Touching			
**Vertebral ridge**	Weakly segmented			Weakly segmented				Non-segmented			
**Rib nodules**	Prominent, 14–15			Prominent, 15				Large, prominent, 15–16			
**Ground color**	Dark brown to black			Blackish				Blackish			
**Color of light markings**	Dark-orange to red			Bright-orange				Orange to dark-red			
**Location of light markings**	Head, vertebral ridge, rib nodules, vent, whole limbs and tail			Head, vertebral ridge, rib nodules, vent, whole limbs and tail				Head, vertebral ridge, rib nodules, vent, dorsal surface limbs, ventral tail ridge			
**Lateral grooves on tail**	Absent			Absent				Absent			

Character abbreviations**:** SVL: Snout-vent length; RHL: Relative head length; RHW: Relative head width; RIND: Relative internarial distance; RAGD: Relative axilla-groin distance; RTRL: Relative trunk length; RTAL: Relative tail length; RVL: Relative vent length; RFLL: Relative forelimb length; RHLL: Relative hindlimb length; RMXHW: Relative maximum head width; RVTW: Relative vomerine tooth series width; RVTL: Relative vomerine tooth series length. M: Male. F: Female. For abbreviations see “Materials and Methods”.

**Eggs and clutch:** The clutch size is unknown. The diameter of ripe eggs in the ovaries of a female paratype (ZMMU A5956) ranged from 1.6 to 1.7 mm (*n*=5, mean=1.7 mm). The animal pole was dark brown and the remaining area of ova was cream.

**Larval morphology:** Description of larval morphology is based on a single larval specimen (ZMMU A5957,Grosse (2013) stage 40) (see Referred specimens for details). Details of larval morphology are presented in Figure 6.

Scale bar: 3 mm. Photos by Nikolay A. Poyarkov.

**Larval measurements (all in mm):** SVL 10.5; HL 3.8; HW 3.4; OL 0.8; AGD 5.9; TAL 9.7; FLL 3.6; HLL 2.8; MXTAH 2.6.

**Larval external morphology:** Body elongated, higher than wide. Head large, trapezoidal in shape, wide and slightly depressed with short, flattened snout, comprising 37% of snout-vent length (SVL), gently sloping in lateral view, two times wider than body in dorsal view. Snout truncate in dorsal view (Figure 6B), rounded in lateral view (Figure 6A). Tail subequal to body length comprising 93% of SVL; myotomes on body and tail not discernable in lateral view. Nostrils rounded, small, oriented anterolaterally, located much closer to snout tip than to eye. Eyes large, rounded, with lateral orientation but still visible in dorsal view (Figure 6B). Limbs thin, forelimbs longer than hindlimbs, HLL/FLL ratio 79.1%. Forelimbs with four well-developed elongated fingers; forelimb turned, palm facing ventrally; relative finger lengths: 4FL<3FL<1FL<2FL. Hindlimbs with knee joint already formed and four well-developed toes, fifth toe as nub; relative toe lengths: 5TL<4TL<3TL<1TL<2TL. Orbit diameter (OL) 7.7% of SVL. Short longitudinal slit for vent. Height of tail musculature at highest portion comprises 50%–60% of tail height. Maximum height of dorsal tail fin 45%–55% of maximum tail height. Ventral tail fin two times lower than dorsal tail fin. Ventral tail fin roughly at level of vent, dorsal fin lower than head, at level of axilla and reaching maximum height mid tail. Tail tip sharply pointed (Figure 6A). Skin completely smooth; lateral line organs visible on ventral side of head; mouth open with well-developed teeth; no remains of yolk (Figure 6C); gills well-developed, much higher than body, with fimbriae clearly visible.

**Larval coloration in life:** In life body background color ochre to golden (Figure 6A, B), ventral surfaces pinkish, translucent (Figure 6C). Body, tail, and head pigmented dorsally: tail almost uniform purple-gray with rare golden speckles, pigmentation forms tortoise-shell golden–dark-gray pattern on body and dorsal fin, head pigmentation less dense, reduced to grayish spots and dots. Few dark spots on ventral fin and limbs. Eyes, except for pupil, fully pigmented, iris golden (Figure 6A).

**Position in mtDNA genealogy and sequence divergence:** According to our mtDNA data, *Tylototriton kachinorum* **sp. nov.** belongs to clade 1 of the subgenus *Tylototriton* s. str. (Figure 2) and is grouped with *Tylototriton* species from the Shan Plateau of Myanmar (*T. shanorum*, *T. ngarsuensis*) and Himalaya (*T. himalayanus*). Uncorrected genetic *P*-distances between *Tylototriton kachinorum* **sp. nov**., 16S rRNA sequences, and all homologous sequences of congeners available included in our analyses varied from 5.3% (with sister species *T. himalayanus*) to 14.6% (with *T. lizhenchangi*) (Table 2).

**Distribution and biogeography:** To date, *Tylototriton kachinorum* **sp. nov.** is known only from a single locality on the slopes of Ingyin Taung Mountain, Kachin State, Myanmar (Figure 1) at elevations from 900 to 1 050 m a.s.l. The Ingyin Taung Mountain belongs to the southernmost part of the Kachin Hills – a heavily forested group of highlands in the extreme northeastern area of Kachin State, consisting of a series of mountain ranges running mostly in a north-to-south direction. The actual distribution of *Tylototriton kachinorum* **sp. nov.** may be wider: it is anticipated that the new species occurs in the montane forests of adjacent mountains surrounding the largest inland lake of Kachin State, Indawgyi Lake, and possibly further northwards along the Kumon Bum subrange of the Kachin Hills.

**Figure 7:**
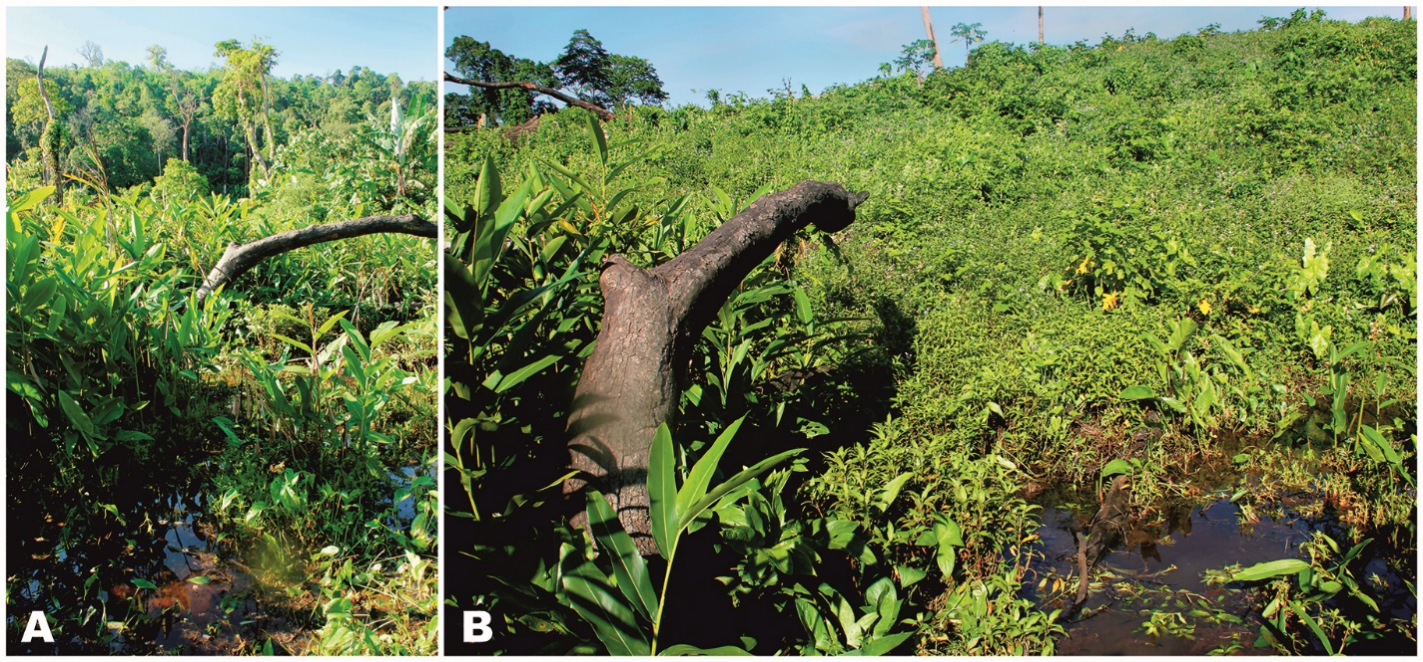
Breeding habitats of *Tylototriton kachinorum *sp. nov. at type locality

**Natural history notes:** Our knowledge on the biology of *Tylototriton kachinorum* **sp. nov.** is scarce. Adult animals were encountered at night after 1900 h in flooded areas of shallow slow-moving streams and artificial ponds in forest clearings, which were used by local Kachin farmers as a watering place for cattle (Figure 7). Surrounding areas were covered by secondary bamboo forest and primary mixed evergreen tropical forest. Adult male newts were observed slowly moving along the clay bottom in clear water 20–40 cm deep; both females and the larval specimen were collected in deeper areas (60–100 cm deep) from dense water vegetation using a dip-net. Courtship behavior of male newts was observed in July. Local Kachin farmers reported that they often find adult newts walking in dense vegetation far from waterbodies, especially after rain; they are also often encountered in wells they construct. The new species is known to local Kachin people as “*Lan Yan*” literally meaning “water agama” in their native language.

Temporary swamps in forest clearings within montane tropical forest on Ingyin Taung Mountain, Indawgyi Lake area, Kachin State, Myanmar, where larva and adult specimens were collected. Photos by Parinya Pawangkhanant.

Other species of amphibians recorded syntopically with the new species at the type locality include *Microhyla heymonsi* Vogt, *Microhyla mukhlesuri* Hasan, Islam, Kuramoto, Kurabayashi & Sumida, *Microhyla butleri* Boulenger, *Limnonectes limborgi* (Sclater), *Limnonectes* sp., *Fejervarya* sp., *Kurixalus* sp., *Feihyla vittata* (Boulenger), *Rhacophorus bipunctatus* Ahl, *Polypedates mutus* (Smith), and *Raorchestes parvulus* (Boulenger).

**Comparisons:** According to phylogenetic analyses, *Tylototriton kachinorum* **sp. nov.** falls into clade 1 of the subgenus *Tylototriton* s. str. and morphological comparisons with members of this group appear to be the most pertinent. The new species can be easily distinguished from members of the subgenus *Yaotriton* (clades 3–5 in Figure 2) by having light color markings on parotoids, lips, vertebral ridge, rib nodules, limbs, and ventral tail ridge (vs. dark body coloration except for palms and soles, vent region, and ventral ridge of tail in most members of the subgenus *Yaotriton*, with the exception of *T. panhai*). The new species can be further distinguished from *T. panhai* by having light color markings on entire limbs (vs. distinct light markings only on palms, soles, and fingers).

Morphometric comparisons and morphological differences in several diagnostic characters between *Tylototriton kachinorum* **sp. nov.** and the closely related species of the subgenus *Tylototriton* are summarized in Table 4. In particular, the new species can be distinguished from *T. taliangensis* (in clade 2 of the subgenus *Tylototriton*, sometimes regarded as a separate subgenus or full genus *Liangshantriton*; see Gong et al., 2018) by having distinct rib nodules, light markings on rib nodules, lips, and parotids (vs. lack of distinct rib nodules, mostly dark charcoal-black body coloration with light orange to red markings only on posterior part of parotoids, digits, palms, soles, vent, and ventral tail ridge). From *T. pseudoverrucosus* (clade 2) and *T. kweichowensis* (clade 1 of the subgenus *Tylototriton*, sometimes regarded as a separate subgenus *Qiantriton*), *Tylototriton kachinorum* **sp. nov.** can be distinguished by having isolated light markings on rib nodules (vs. connected markings forming light dorsolateral lines).

*Tylototriton kachinorum* **sp. nov.** can be distinguished from *T. uyenoi*, *T. pulcherrimus*, *T. shanjing*, and *T. yangi* by having dull orange-brown to yellowish-brown light markings (vs. much brighter orange to bright-yellow light markings). In particular, *T. pulcherrimus* has a series of bright-orange glandular spots located ventrolaterally and on flanks (vs. dark-brown coloration of flanks lacking light spots in new species); *T. yangi* has contrasting charcoal-black coloration of head and lips with only posteriormost part of parotoid colored bright orange, and no light ventral markings on body and tail (vs. all head dull orange-brown with slightly lighter lips and parotoids, light markings present on ventral tail ridge and vent in new species). *Tylototriton kachinorum* **sp. nov.** can be distinguished from *T. uyenoi* by relatively longer head in males (RHL 27.6 vs. 24.7), greater internarial distance (RIND 7.7–8.0 vs. 7.0–7.1 for both sexes), and longer tail in both sexes (RTAL 120.5 vs. 115.0 for males; 100.5–114.0 vs. 88.0–97.0 for females) (Table 4). Males of the new species can be further diagnosed from males of *T. shanjing* by having comparatively wider head (RHW 25.1 vs. 22.2), longer tail (RTAL 120.5 vs. 104.4), greater internarial distance (RIND 7.7 vs. 7.1), comparatively wider (RVTW 9.0 vs. 6.7) and longer vomerine tooth series (RVTL 12.8 vs. 8.8) (Table 4). The new species can be further differentiated from *T. shanjing* by non-segmented vertebral ridge (vs. well-segmented) and brown to dark-brown background coloration of body (vs. blackish background coloration).

*Tylototriton kachinorum* **sp. nov.** can be distinguished from *T. verrucosus* by having light ventral markings on body and tail (vs. no ventral markings on body and tail), comparatively longer head (RHL 27.6 vs. 24.3 in males, RHL 23.9–24.9 vs 21.7 in females), comparatively wider head in both sexes (RHW 25.1–25.4 vs. 20.5–23.7), greater internarial distance in both sexes (RIND 7.7–8.0 vs. 6.2–7.0), longer tail in both sexes (RTAL 120.5 vs. 104.9 for males; 100.5–114.0 vs. 102.5 for females) (Table 4), non-segmented vertebral ridge (vs. well-segmented), and brown to dark-brown background coloration of body (vs. blackish background coloration).

The new species can be distinguished from *T. podichthys* by having comparatively shorter head in both sexes (RHL 23.9–27.6 vs 28.1–34.3), much longer tail in both sexes (RTAL 100.5–120.5 vs. 79.2–104.8), truncate snout (vs. rounded), comparatively smoother skin on parotoids and dorsal surface of head, comparatively longer limbs (limbs widely overlap when adpressed to body in the new species vs. digit tips touch when limbs are adpressed to body in *T. podichthys*), 13–14 rib nodules (vs. 15–16 rib nodules), and duller coloration with orange-brown light markings and brown to dark-brown background (vs. orange to dark-red light markings and blackish background) (Table 4).

*Tylototriton kachinorum* **sp. nov.** can be distinguished from *T. anguliceps* by having larger body size in both sexes (SVL 68.6±2.9 mm in males and 72.5–84.8 mm in females of new species vs. 61.1–62.5 mm and 70.6±3.4 mm in *T. anguliceps*), comparatively wider head in both sexes (RHW 25.1–25.4 vs. 22.7–23.4), greater internarial distance in both sexes (RIND 7.7–8.0 vs. 6.5–7.4), longer tail in both sexes (RTAL 100.5–120.5 vs. 91.2–102.3), and comparatively wider (RVTW 8.8–9.0 vs. 6.4–7.8) and longer vomerine tooth series (RVTL 11.4–12.8 vs. 9.2–10.8) (Table 4). The new species can be further diagnosed from *T. anguliceps* by having wide protruding supratemporal ridges (vs. narrow and steep supratemporal ridges), very small and almost indiscernible sagittal ridge (vs. long and notably protruding sagittal ridge), wide and non-segmented vertebral ridge (vs. narrow and weakly segmented vertebral ridge), less distinct 13–14 rib nodules (vs. rib nodules more distinct and protruding, not less than 15), and duller coloration with orange-brown light markings and brown to dark-brown background (vs. bright-orange light markings and blackish background) (Table 4).

Phylogenetically and morphologically, *Tylototriton kachinorum* **sp. nov.** is most closely related to other species of *Tylototriton* inhabiting Myanmar (e.g., *T. shanorum* and *T. ngarsuensis*) from the Shan Plateau and *T. himalayanus* from Nepalese Himalaya (Table 4). The new species can be readily distinguished from *T. shanorum* by having longer head in males (RHL 27.6 vs. 22.4), greater internarial distance in both sexes (RIND 7.7–8.0 vs. 6.0–6.7), notably longer tail in both sexes (RTAL 120.5 vs. 111.2 in males, 100.5–114.0 vs. 97.0–97.8 in females), wider vomerine tooth series in both sexes (RVTW 8.7–9.0 vs. 6.1), longer vomerine tooth series in males (RVTL 12.8 vs. 10.7) (Table 4), supratemporal bony ridges beginning at anterior corner of orbit (vs. supratemporal bony ridges beginning at loreal region), and wide, non-segmented vertebral ridge (vs. narrow, weakly segmented vertebral ridge). *Tylototriton kachinorum* **sp. nov.** has generally lighter coloration than *T. shanorum*: brownish ground color with orange-brown light markings (vs. more contrasting dark brown to black background color with yellow to reddish-brown light markings) and light markings present only on ventral tail ridge (vs. lateral sides of tail with light markings) (Table 4).

*Tylototriton kachinorum* **sp. nov.** can be easily distinguished from *T. ngarsuensis* by having generally smaller body size in males (SVL 68.6±2.9 mm vs. 74.9–76.4 mm) and females (SVL 72.5–84.8 mm vs. 102.3 mm), comparatively longer head in males (RHL 27.6 vs. 24.0–26.0) and females (RHL 23.9–24.9 vs. 21.5), longer tail in males (RTAL 120.5 vs. 98.0–103.5), generally longer tail in females (RTAL 100.5–114.0 vs. 104.6), and comparatively shorter vent length (in males RVL 10.2 vs. 10.7–12.3, in females 5.6–6.8 vs. 8.0) (Table 4). The new species can be further distinguished from *T. ngarsuensis* by having truncate snout (vs. rounded snout), supratemporal ridges starting at anterior corner of orbit (vs. supratemporal bony ridges beginning posterior to orbit), non-segmented vertebral ridge (vs. weakly segmented), and 13–14 weakly distinct rib nodules (vs. 15 well-distinct rib nodules) (Table 4). *Tylototriton kachinorum* **sp. nov.** also has much lighter and duller colorationthan *T. ngarsuensis*: background color brown to light brown (vs. nearly black) with light orange-brown markings on rib nodules, parotids, and whole limbs (vs. no light markings on rib nodules or parotids, dark-yellow coloration present only on palms and soles) (Table 4).

Morphologically, *Tylototriton kachinorum* **sp. nov.** most resembles its sister species, *T. himalayanus* from Nepal; however, it can be readily distinguished by the following morphological attributes: comparatively longer head in males (RHL 27.6 vs. 24.5), notably wider head in both sexes (RHW 25.1–25.4 vs. 23.0–23.6), shorter internarial distance in both sexes (RIND 7.7–8.0 vs. 8.2–8.4), and longer tail in both sexes (RTAL 100.5–120.5 vs. 98.0–100.6) (Table 4). The new species has generally smoother skin than *T. himalayanus* and can be distinguished from the latter species by having weakly distinct 13–14 rib nodules (vs. large and prominent 16 rib nodules) and absence of lateral grooves on tail (vs. very distinct). Coloration of the new species is similar to *T. himalayanus*, but light markings are also present on labial regions, parotoids, rib nodules, and whole limbs (vs. no light markings on head and rib nodules, on limbs only on ventral surfaces) (Table 4).

**Etymology:** The specific name “*kachinorum*” is a Latin adjective in the genitive plural (masculine gender), derived from the name of the Kachin people who inhabit the montane areas of northern Myanmar and adjacent territories (Kachin Hills), including the type locality of the new species.

**Recommended vernacular name:** We recommend the following name in English: *Kachin Crocodile Newt*. Recommended vernacular name in Burmese (Myanmar) language: *Kachin Yae Poke Thin*.

**Conservation status:** *Tylototriton kachinorum* **sp. nov.** is, to date, known from a single locality in the southern part of the Kachin Hills of northern Myanmar; the actual range of the new species is unknown. The new species is anticipated to inhabit elevations above 900 m a.s.l. on mountains surrounding the Indawgyi Lake valley and may be found in other parts of the Kachin Hills. Further research is required to estimate the actual distribution, population trends, and possible threats to the new species. *Tylototriton kachinorum* **sp. nov.** appears to be associated with montane forests and may be affected by growing anthropogenic pressure and forest destruction observed in different areas of Kachin State in Myanmar. Given the available information, we suggest *Tylototriton kachinorum* **sp. nov.** to be tentatively considered as a Vulnerable (VU) species following IUCN’s Red List categories (IUCN, 2001).

## DISCUSSION

Our phylogenetic data largely confirmed the phylogeny of *Tylototriton* as presented in the earlier studies of Phimmachak et al. (2015), Khatiwada et al. (2015), Wang et al. (2018), and Grismer et al. (2018a). In particular, our data support the subdivision of *Tylototriton* into two major groups, traditionally regarded as subgenera: *Tylototriton* s. str. (clades 1 and 2) and *Yaotriton* (clades 3–5). Two other subgenera recently proposed by Fei et al. (2012), namely *Qiantriton* and *Liangshantriton* are nested within the radiation of *Tylototriton* s. str. (Figure 2) and possibly should not be regarded as independent subgenera or even genera (see Gong et al., 2018; Hernandez, 2016). Our mtDNA-based genealogy is insufficiently resolved in a number of deeper tree nodes; however, it unambiguously suggests that clade 1 of *Tylototriton* s. str. is subdivided into three main subclades with prominent geographic structuring. The northernmost member of this group – *T. kweichowensis*, occurring in the Guizhou Plateau of China (Figure 1, samples 7–10), is distant from other members of clade 1 and its phylogenetic position is unresolved. Most other species of clade 1, which occur in northern Indochina and Yunnan Province of China, form a monophyly (including *T. verrucosus*, *T. shanjing*, *T. uyenoi*, *T. anguliceps*, *T. podichthys*, *T. pulcherrimus*, *T. yangi*, and *Tylototriton* sp. 1) (Figure 1, samples 17–18 and 24–48). Finally, four species from the northern part of Myanmar and Himalaya form a distinct monophyletic group (including *T. shanorum*, *T. ngarsuensis*, *T. himalayanus*, and *Tylototriton kachinorum* **sp. nov.**).

Studies on the taxonomic status of *Tylototriton* species in Myanmar have been delayed, even though the genus has been reported from the country for long time (see Gyi, 1969). Nishikawa et al. (2014) assigned *Tylototriton* from the Shan Plateau of eastern Myanmar to a new species, *T. shanorum*, whereas Grismer et al. (2018a) recently demonstrated the presence of divergent lineages of *Tylototriton* within different parts of the Shan Plateau and described a second species for the country – *T. ngarsuensis*. Contrary to the tree of Grismer et al. (2018a), our analysis suggests that *T. shanorum* is paraphyletic with respect to *T. ngarsuensis* and is subdivided into two lineages (Figure 2). However, it is worth noting that the second lineage of *T. shanorum* is based on data from a single specimen, reported by Nishikawa et al. (2014) (KUHE42348), which was obtained through the pet trade and presumed to come from Myanmar. Its assignment to *T. shanorum* is thus tentative and this specimen possibly represents a new lineage of *Tylototriton* from the Shan Plateau, yet undiscovered in the wild. Hence, considering the profound morphological differences between *T. ngarsuensis* and *T. shanorum* reported by Grismer et al. (2018a), though genetic divergence between these species was low (*P*=1.8% for *ND2* gene), we consider that *T. ngarsuensis* indeed represents a distinct species of *Tylototriton*.

Our description of *Tylototriton kachinorum* **sp. nov.** thus represents the third species of *Tylototriton* endemic to Myanmar. *Tylototriton kachinorum* **sp. nov.** is more closely related to *T. himalayanus* than to *T. shanorum* and *T. ngarsuensis*, occurring on the Shan Plateau in the eastern part of the country. This fact can be explained from a biogeographic viewpoint: the Kachin Hills represent the southernmost outcrop of the Great Himalaya ridge (Hadden, 2008) and are isolated from the Shan Plateau by the Irrawaddy (Ayeyarwaddy) River valley, which may serve as an important biogeographic border for forest-dwelling taxa. *Tylototritonhimalayanus*, which can be found in Nepal and possibly along the Great Himalaya Ridge, was previously reported for northern Myanmar (Hernandez, 2016, 2018), but without any information on voucher specimens or reasons for such identification. There is a possibility that these records are based on the misidentification of *Tylototriton kachinorum* **sp. nov.**

The present work indicates that *Tylototriton* diversity in Myanmar is still underestimated. Phimmachak et al. (2015) and Grismer et al. (2018a) reported sequences of *Tylototriton* cf. *verrucosus* from two regions of northern Myanmar: Sagaing Region (sample 18, see Figure 1) and the eastern part of Kachin State (sample 17, see Figure 1). Surprisingly, these populations were found to be distantly related to *Tylototriton kachinorum* **sp. nov.**, despite the geographical proximity to the southern part of Kachin Hills where the new species occurs. Sagaing and eastern Kachin *Tylototriton* populations (indicated herein as *Tylototriton* sp. 1) were reconstructed as members of the *T.verrucosus* species complex; however, they formed an mtDNA lineage clearly distinct from all currently recognized species. These data suggest that *Tylototriton* cf. *verrucosus* from northern Myanmar might represent a currently undescribed species and further morphological and phylogenetic studies are required to evaluate its taxonomic status. In agreement with the results of Wang et al. (2018）, our analysis also shows deep subdivision and the presence of several lineages of possibly full-species status within *T. asperrimus*, *T. dabienicus*, and *T. wenxianensis* of the subgenus *Yaotriton*.

Our study indicates that knowledge on amphibian diversity in montane regions of northern Myanmar is still far from complete and further diversity is likely to be revealed with additional survey efforts. A number of recent studies have shown that species diversity of amphibians and reptiles is widely underestimated across Myanmar (Grismer et al., 2017a, 2017b, 2018a, 2018b, 2018c; Mulcahy et al., 2018), and it is likely that further surveys on this geographically complex and insufficiently studied region will lead to more discoveries. Recent economic development of Myanmar has led to increasing habitat loss and modification across the country (Li & Quan, 2017). Further intensified survey efforts and biodiversity assessments are urgently required for effective conservation management of yet unrealized herpetofaunal diversity in Myanmar.

## NOMENCLATURAL ACTS REGISTRATION

The electronic version of this article in portable document format will represent a published work according to the International Commission on Zoological Nomenclature (ICZN), and hence the new names contained in the electronic version are effectively published under that Code from the electronic edition alone (see Articles 8.5–8.6 of the Code). This published work and the nomenclatural acts it contains have been registered in ZooBank, the online registration system for the ICZN. The ZooBank LSIDs (Life Science Identifiers) can be resolved and the associated information can be viewed through any standard web browser by appending the LSID to the prefixhttp://zoobank.org/.

### Publication LSID

urn:lsid:zoobank.org:pub:FC2F997C-2E7B-4B22-9376-E335E7A84AB1.

### *Tylototriton kachinorum* LSID

urn:lsid:zoobank.org:act:FF992F77-436E-4164-A1C2-3EB8D220648F.

We thank the Ministry of Natural Resources and Environmental Conservation Forest Department for the collection and export permits and the staff of the Indawgyi National Park for help with organization of fieldwork. We thank the staff of the guesthouse in Lon Ton Village for their hospitality. N.A.P. thanks Duong Van Tang and Anna S. Dubrovskaya for help during lab work and to Evgeniy Popov for help with map design. For permission to study specimens under their care and permanent support, we thank Valentina F. Orlova (ZMMU), Roman A. Nazarov (ZMMU), and Konstantin D. Milto (ZISP). We thank Natalia Ershova for proofreading. We are sincerely grateful to L. Lee Grismer and Bryan Stuart for their kind help and useful comments, which helped us to improve the previous version of this manuscript.
